# mHealth: Potentials and Risks for Addressing Mental Health and Well-Being Issues Among Nepali Adolescents

**DOI:** 10.3389/fpubh.2021.563515

**Published:** 2021-04-23

**Authors:** Siobhan K. Yilmaz, Alok K. Bohara

**Affiliations:** Department of Economics, University of New Mexico, Albuquerque, NM, United States

**Keywords:** sociocultural, developing country, economic development, technological change, health, quantitative methods, human development, innovation

## Abstract

Adolescents are slowly being recognized as a generation, worldwide, that may require different policy approaches to improve staggering statistics on their failing well-being, including mental health. By providing the support to allow the next generation to achieve better mental health outcomes, they are going to be more economically successful and the future economic growth of nations can be better assured. Adoption of mobile-based health interventions (e.g., mHealth) has garnered a lot of attention toward this end. While mHealth interventions are growing in popularity, many researchers/policy-makers appear to have neglected assessing potential (indirect) costs/negative consequences from their use. Evidence from the developed world shows strong associations between extensive cell phone use and negative mental health outcomes, but similar research is minimal in developing world contexts. Additionally, the bulk of work on the outcomes of mobile phone use is studied using a unidirectional approach with blinders to front-end motivations. Using primary data from a large-scale, school-based survey of older adolescents in southwestern Nepal (*N* = 539), this work investigates such a tension between mobile/smartphone usage as a true mobile health (mHealth) opportunity in Nepal or as a potential problem, introducing additional deleterious well-being effects from over-use. Founded in Basic Psychological Needs Theory (BPNT), robust results of analyses using full structural modeling approaches (and traditional regression-based sensitivity analyses) indicate support for the BPNT framework in explaining statistically significant positive associations between bullying and anxiety, as well as, negative associations between bullying and grit, including evidence to support the mediating role of problematic mobile phone use in these relationships. More than 56% of the sample showed indicators of mild to moderate anxiety and over 10% claim experiences of bullying, coupled with over 75% of the sample scoring above the midline of a problematic mobile phone use scale, all of which motivates the relevance of our findings. Potential policy implications of these findings, and mention of other intriguing avenues for future work are further discussed.

## Introduction and Background

Of the 1.2 billion people under age 18 across the globe, 90% of them live in the developing world (1). The economic future of the world will depend on this next generation and improvements to their current well-being trajectories (2, 3). Estimates indicate 20–50% of young people in the Southeast Asia (SEA) region report being bullied, 7.1% are using alcohol, 9.7% are smoking, 8.4% report being lonely most or all of the time, 6.9% report being so anxious they lose sleep, and 6.8% have admitted to seriously considering suicide within the prior year (4). Such behavioral and mental health/well-being concerns are exacerbated by governments and healthcare systems which either do not acknowledge the importance of mental health, fail to address adolescents differently than younger children or adults, or both.

As chronic conditions, such as mental health, continue to gain attention, mobile-based health initiatives (or mHealth) are one potential solution to address the lack of attention, cost, and availability of healthcare professionals, particularly within developing world contexts (5, 6). Although limited in scale, most existing evidence of the acceptability and efficacy of mHealth approaches to change health-related behaviors has been well-received (7, 8), although thorough assessment of true economic benefits is still lacking (9). Due to their existing high use of mobile-based technologies, the use of mHealth approaches in adolescent populations may be additionally promising (10–12). However, while use of mobile technology is prolific among adolescent populations, there is also evidence of lack of sufficient research fully accounting for the effectiveness and potential consequences of pursuing mHealth initiatives (13, 14).

Overuse/maladaptive uses of mobile/smartphones in the developed world has been strongly associated with numerous negative health/well-being outcomes (15, 16). However, there is limited work in the developing world regarding such impacts, with most focused on India (17, 18). Initial findings, however, do appear consistent with those from the developed world, which implies a very bleak picture for adolescent well-being if mobile phones are seen as the “solution” to an already suffering demographic.

When examined with empirical rigor, though, this outlook may not be so bleak. There is other work indicating that it is only those adolescents most prone to suffer from behavioral, mental, and emotional struggles who are going to be drawn to overuse mobile phones. However, determination of directionality of such relationships is fraught with a lack of clarity. Lack of focus on motivating factors may explain part of this. Incorporating theoretical frameworks from fields such as Positive Psychology and Sociology may allow for a clearer understanding, while also analyzing data with the empirical rigor associated with a field such as Economics. In that vein, this work uses primary data from secondary school students in urban and rural Nepal to assess the connections between life burdens, mobile phone overuse/maladaptive use, and overall well-being of adolescents through estimating a full structural model based on the assumptions of Basic Psychological Needs Theory (BPNT).

### Research Context: Nepal

Nepal is one of the poorest and neediest regions in terms of addressing adolescent well-being. Of the 11.8% of the population estimated to be between 13 and 17, over 50% of adolescents report being bullied, making this the highest rate in the SEA region (4). Additional burdens adolescents, and in particular females, face in this country include high levels of gender-based violence/inequity (e.g., domestic violence, menstrual taboos, child marriage), which have damaging mental and physical health consequences (19–21). A healthcare treatment system that faces infrastructure and treatment malfunction, with limited access to mental health care, only exacerbates such situations. Nepal only has 2.1 physicians per 10,000 people (the United States has 24.5) (22), and stigma surrounding mental health problems is high.

Indicative of the cumulative effect of such burdens is that Nepal has the second highest adolescent suicide rate regionally. A recent WHO Report (23) found 14% of surveyed adolescents thought about/planned suicide and 10% attempted suicide within the prior 12 months. Such risks also appear to continue on into adulthood, with Nepal having one of the top five (ten) suicide rates globally for women (men) (22). The need is real for interventions aimed at reducing such well-being-damaging burdens and providing treatment/support for them, particularly among adolescent populations.

Unfortunately, most of the existing research/interventions addressing these areas in Nepal has primarily focused on the plight of former child soldiers and conflict/displaced youth (24, 25). Furthermore, the use of mHealth interventions, while growing, is still in its infancy in Nepal, primarily focusing on more standard telehealth and tracking functionalities (26, 27). At the same time, cell phone/smartphone adoption is growing rapidly in Nepal, with 70–90% of households owning mobile phones (20). The World Bank (23) also reports that mobile-cellular telephone subscriptions per 100 people in Nepal jumped from 34.4 to 96.7% between 2010 and 2015.

### Prior Literature on Mobile Phones and Negative Well-Being

With the growth in development, innovation, and adoption of mobile/smartphone technology, there is also a growth in research surrounding the consequences of its overuse. Along with findings supporting that high mobile phone use is linked with lost sleep (28–30), there is consistent evidence of higher associations to anxiety, stress, and depression (31–33).

The empirical limitation in these analyses is that direction of causality has yet to be well-established. While there is work that has focused on theoretical explanations of the motivation for mobile phone use (34), such work is not supported with empirical validation. The general assumption in empirical work is that cell phone use is the predecessor of negative mental health/well-being, supported by laboratory experiments (35, 36). Several long-term studies have also confirmed this (37, 38), along with strong indications of bi-directionality (39, 40), which may imply: the rich are getting richer (or in this case, the depressed are getting more depressed, due to their over-use of mobile phones).

To bring the literature forward with respect to further clarity about the relationships indicated, while also accounting for motivational elements that partially explain the findings already seen, this work examines the tension between mobile phone/smart phone usage as a true mHealth opportunity in Nepal and its potential for introducing deleterious effects on well-being from over-use of mobile phones. The impacts of technology on health deserve to be analyzed with the empirical rigor associated with Economics, given that the impacts of health on micro and macro-level economic problems is becoming ever more apparent. Therefore, by using robust empirical techniques we are able to provide additional validity to the conclusions and policy recommendations we make.

## The Model: Framework, Specification, and Hypotheses

### Conceptual Framework

Determining what motivates behavior is among the most central of all social science questions. Within sociology, consideration of social contexts has often served as the jumping-off point to answer this question. Within the study of Self-Determination Theory (SDT), there is the sub-theory of BPNT, which claims that satisfaction and/or frustration of the three needs of autonomy, competence, and relatedness serve as strong motivating factors in many behavioral relationships (41), many of which have well-being outcomes (42, 43).

**Figure 1:**
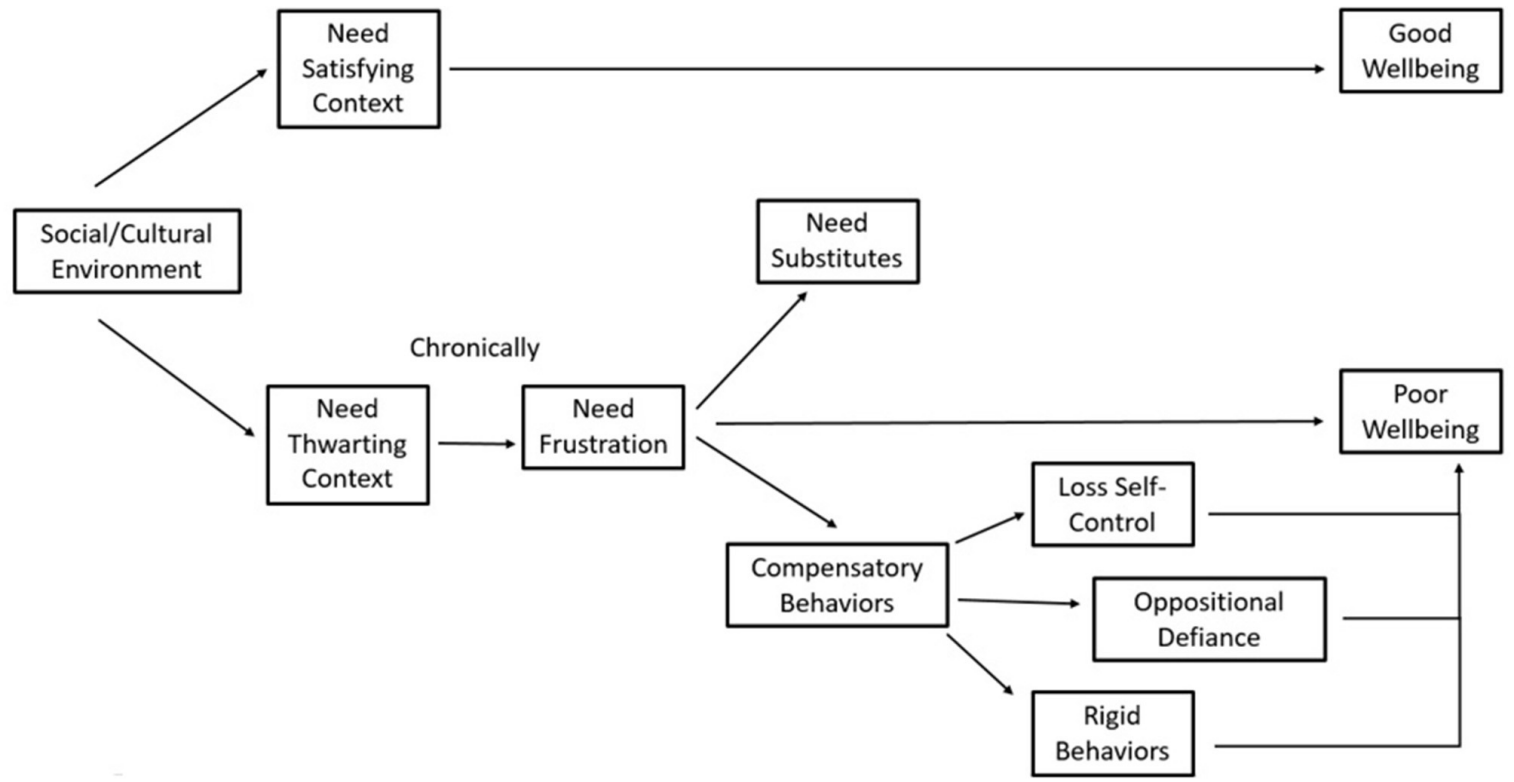
Framework of basic psychological needs theory (BPNT).

As shown in Figure 1, low need satisfaction over time can result in costs, but this deterioration process will be exacerbated when needs are actively “frustrated” (or thwarted). When needs are frustrated, there are two consequences: immediate costs of reduced well-being and chronic need-thwarting leading to the development of coping strategies such as “Need Substitutes” and compensatory behaviors. However, most evidence indicates that the latter may ultimately be short-lived in producing feelings of need satisfaction, and can lead to (or include) further negative outcomes such as anxiety and substance abuse (44, 45).

**Figure 2:**
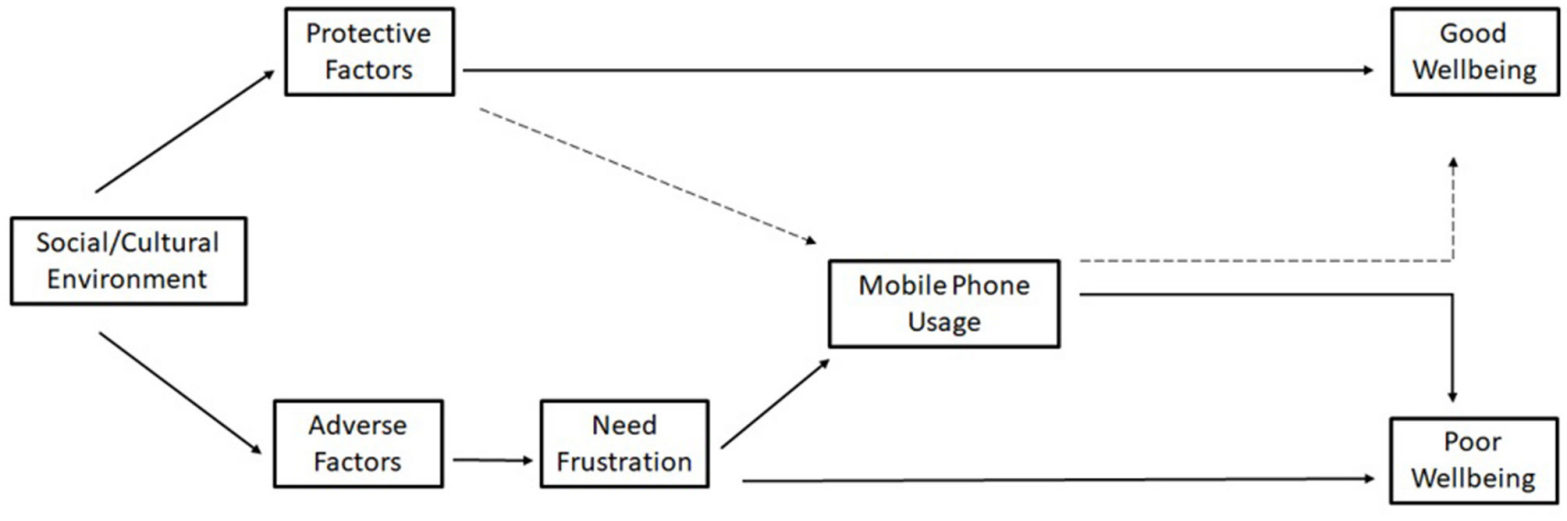
Research application of BPNT framework.

We believe the relationship between problematic mobile phone use (PMPU) and well-being outcomes may follow this theoretical pathway, as seen in Figure 2. The social/cultural context adolescents face includes both protective and adverse environments/pressures which either meet or frustrate the three needs. When needs are frustrated, BPNT indicates that adolescents may seek out remedies to alleviate their sense of loss, which we postulate is facilitated through mobile phones. Use of technology that (attempts) to fulfill psychological voids, however, may be more prone to addiction (46, 47) and associated poorer well-being. Thus, if mobile phones are a medium through which the types of coping strategies BPNT mentions are facilitated, then they have the potential to exacerbate existing states of negative well-being, in congruence with existing literature.

### Empirical Specification

In accordance with the framework and pathways above, we identify the empirical specification below:

(1) $$\begin{array}{l} Anx_{i}=\alpha_{0}+\alpha_{1}PMPU_{i}+\alpha_{2}Bully_{i}+\alpha_{3}AP_{i}^{*}\\ +\alpha_{4}FE_{i}^{*}+\alpha_{5}SS_{i}^{*}+\alpha_{6}X_{1i}+\alpha_{7}X_{2i}+\alpha_{8}X_{3i}+u_{1i} \end{array}$$

(2) $$\begin{array}{l} Grit_{i}=\beta_{0}+\beta_{1}PMPU_{i}+\beta_{2}Bully_{i}+\beta_{3}AP_{i}^{*}\\ +\beta_{4}FE_{i}^{*}+\beta_{5}SS_{i}^{*}+\beta_{6}X_{1i}+\beta_{7}X_{2i}+\;\beta_{8}X_{3i}+u_{2i} \end{array}$$

(3) $$\begin{array}{l} PMPU_{i}=\lambda_{0}+{\lambda_{1}Bully}_{i}+\lambda_{2}AP_{i}^{*}+\lambda_{3}FE_{i}^{*}\\ +\lambda_{4}SS_{i}^{*}+\lambda_{5}PC_{i}+\lambda_{6}{PC2}_{i}+\lambda_{7}FPMPU_{i}\\ +\;\lambda_{8}X_{1i}+\lambda_{9}X_{2i}\;+\;X_{3i}+u_{3i} \end{array}$$

*Anx*_*i*_ and *Grit*_*i*_ represent the well-being outcomes of anxiety and grit^1^ for individual *i*. *PMPU*_*i*_ is a measure of mobile phone overuse/maladaptive use, and *Bully*_*i*_ represents our key need-thwarting factor of bullying experiences. The remaining socio-cultural environmental pressures we consider/control for are academic pressure ($AP_{i}^{*}$), family environment ($FE_{i}^{*}$), and social support *(*${SS}_{i}^{*}$), all latent constructs. In Equation (3), *PC*_*i*_ is the monetary cost of individual *i*'s mobile phone, with *PC2*_*i*_ its square. Friend's PMPU is represented by *FPMPU*_*i*_. Demographic controls for being female, age, and rural area residence are accounted for by *X*_1*i*_*-X*_3*i*_, respectively. White noise error terms for each equation are denoted by u_i_, with the appropriate equation referenced in subscript.

### Hypotheses

Bullying can be staggering and have dramatic impacts on mental/physical health (53, 54), whereas individuals with greater support across environments (including school) show a number of positive outcomes including subjective well-being and lower stress levels (55, 56). In the absence of real-life support, many adolescents may turn to other means, such as media entertainment, to try and meet their needs. Given associations between psychological need frustration and dependency in adolescents (44), adolescents using media (often accessed via mobile phones) for such means would be expected to then have more problematic use of digital technology. This supports the “needs-as-motive” hypothesis among young adults, wherein the trio of needs not only sustains well-being, but also motivates remedial behavior when missing (57). Given strong evidence that coping strategies are ultimately ineffective/maladaptive (45, 58), this would imply further reductions in overall well-being.

Given such evidence, we formally hypothesize:

***Hypothesis #1:*** *Those adolescents bullied more will have worse well-being outcomes – higher anxiety (Anx) and lower grit (Grit)*.

***Hypothesis #2:*** *Those adolescents who are bullied more (Bully) will have more problematic mobile phone use (PMPU)*.

***Hypothesis #3:*** *There will be a significant indirect effect of bullying (Bully) on well-being outcomes (Anx and Grit), acting through problematic mobile phone use (PMPU)*.

Additionally, it is also important to establish any potential relationship to be had between our well-being outcomes. The small literature addressing anxiety and grit consistently indicate a negative relationship (59, 60), including in developing world contexts (61, 62). Thus, we finally hypothesize:

***Hypothesis #4:*** *There will be a negative correlation between anxiety (Anx) and grit (Grit)*.

## Data and Variables

### Data

Data comes from a self-report survey administered to 11 and 12th grade-equivalent adolescents in the urban city of Siddharthanagar and surrounding areas of Pulpa, Gulma, and Argankhachi, in southwestern Nepal. Following an exploratory focus group assessment conducted in the same area prior to this study, which addressed mental health knowledge/awareness, current mobile phone practices/uses, and cultural/developmental burdens seen as most detrimental, the survey used in this study was translated by a professional translator, certified by the local Notary Public Council. The translated survey was further read through by several of the Nepali enumerators and support staff running the survey collection on the ground, prior to its implementation, to ensure that it was conceptually sound in its translation. The survey/data collection was performed on-sight at schools during school time. We *a priori* aimed to collect data from around 600 observations and were only limited by the fixed amount of sampling provided by headmasters at the schools. These goals were met and once restricting our sample to only those adolescents who owned a phone, our final sample size was 539 adolescents. This study was approved by the institutional review board (IRB) of our institution and the executive management group of the Lumbini Center for Sustainability at its Pratiman Neema College venue in Siddharthanagar, Rupandehi district, who also oversaw the collection and supervision of the data collection.

### Key Variables

Well-being outcomes were measured using previously validated survey instruments. *Anx* is based on a modified version of the Beck Anxiety Inventory (BAI) (63), assessing the extent of anxiety-related symptoms such as dizziness and breathing difficulties over the prior 2 weeks. Adjustments were made to adhere to recommendations from a previous validation in a Nepali context (64), which indicated a specificity and sensitivity around 0.90. The final (continuous) measure is composed of the sum of seventeen, 4-point scale items, where a higher score indicates poorer well-being. Summary statistics of this and all other variables can be found in Table 1.

**Table 1:** Summary statistics of variables.

**Variables**	**Description**	**Mean**	**Standard deviation**	**Min/Max**
**Outcome variables**				
*Anxiety (A)*	Validated instrument^†^ addressing various symptoms of anxiety, calculated as the sum of 17 Likert-scored questions (0–3 points). Higher score indicates more symptoms of anxiety	13.38	7.97	0/48
*Grit Score (G)*	Validated instrument^‡^ which captures elements of passion and persistence in life, calculated as the sum of ten, 5-point Likert scale items, divided by 5; Higher score indicates more Grit	3.26	0.49	1.8/5
**Explanatory variables**				
**Key Explanatory Variables**				
*Problematic Mobile Phone Use (PMPU)*	Validated instrument^§^ addressing indicators of addictive tendencies toward mobile phone use, calculated as the sum of 27 5-point Likert scale items, where a higher score indicates more problematic usage	86.65	21.07	27/135
*Bullying (B)*	Sum of three binary indicators related to bullying pressures	0.361	0.713	0/3
	Physically Hurt Prior Year	0.138	–	0/1
	Bullied at School	0.107	–	0/1
	Bullied Outside School	0.116	–	0/1
**Socio-Cultural Environmental Pressures Controlled For**				
Academic Pressures (AP^*^)	Latent construct measured by four, 5-point Likert questions detailing pressures from the school environment (1=Strongly Disagree, 5=Strongly Agree)			
	Worry About Exam Scores	4.01	1.30	1/5
	Teachers Too Controlling	3.67	1.39	1/5
	School Competitive	3.85	1.29	1/5
	School Success is Life Success	4.12	1.28	1/5
Family Environment (FE^*^)	Latent construct measured by four, 5-point Likert questions detailing violence and control in the home (1=Strongly Disagree, 5=Strongly Agree)			
	Parents Check Phone	3.17	1.62	1/5
	Physically Hurt in Home	2.54	1.57	1/5
	Punished for Bad Grades	2.64	1.62	1/5
	Women Tolerate Violence	2.62	1.63	1/5
Social Support (SS^*^)	Latent construct measured by six binary indicators for having someone or somewhere to go to deal with a series of financial or social issues			
	Borrow Money	0.73	–	0/1
	Stay With	0.70	–	0/1
	Confide in About Violence	0.64	–	0/1
	Help with Harassment Situation	0.64	–	0/1
	Place Meet Same Sex Friends	0.44	–	0/1
	Member of Club/Youth Group	0.41		
**Instruments (For PMPU)**				
*Phone Cost (PC)*	Cost of mobile phone in Nepali Rupees divided by 1,000.	19.35	16.43	0.2/110
*Phone Cost Sq. (PC2)*	*Phone Cost* squared	643.62	1469.37	0.04/12100
*Friend's PMPU (FPMPU)*	Reworking of validated instrument*^3^* addressing indicators of closest friend's addictive tendencies toward mobile phone use, calculated as the sum of six 5-point Likert scale items, where a higher score indicates more problematic usage	20.09	5.62	6/30
**Additional controls**				
*Age (X1)*	Age of adolescent in years	17.6	1.18	15/25
*Female (X2)*	=1 if adolescent is female, 0 otherwise	0.51	–	0/1
*Rural (X3)*	=1 if adolescent is from a rural high-school, 0 otherwise	0.48	–	0/1

† **(63) & (64)**.

‡ *(65)*.

§ *(66)*.

*Grit* is the Duckworth Grit Scale (65), which has an internal consistency around 0.80. Grit has previously served as a proxy for positive well-being, including among adolescent/young adult populations (67, 68). The sum of ten, 5-point Likert-scale items is divided by five to obtain the final (continuous) measure used in estimation, where a higher score indicates more grit.

*PMPU* was operationalized through the use of Bianchi and Phillip's (66) Problematic Mobile Phone Use survey. This 27 item, 5-point Likert-scale instrument assesses dimensions of problematic phone use. It has been validated in terms of internal reliability (0.93) and validity in capturing the addictive tendencies associated with maladaptive use. The final (continuous) measure used in estimation is the sum of the 27 items, where a higher score is indicative of more (problematic) use.

To allow for proper identification of our empirical model, we also include the use of three variables believed to influence respondent's PMPU, but not expected to directly influence well-being. In traditional econometric approaches, these would be deemed the “instrumental variables.” *PC* is the cost of the respondent's mobile phone in thousands of Nepali rupees (NR), ascertained from an opened ended question. *PC2* is simply the square of this measure. Use of these variables stems from the assumption that amount of phone usage depends on its cost, with more usage at lower cost levels (69, 70). *FPMPU* is a modified version of (66) survey instrument, rephrasing six of the questions to reflect respondent's perceptions of their closest friend's interactions with his/her mobile phone, where the assumption is that the behaviors of a close friend in relation to his/her phone usage will correlate with those of the respondent's own usage. Summation of these six, 5-point Likert-scale questions produces the continuous measure used in estimation.

*Bully* is the summation of three binary indicators for having been bullied in the prior month outside of school, inside of school, and having been physically hurt by someone at school. Framing and use of these measures is consistent with large-scale national and international work examining adolescent life to determine overall well-being (71).

While bullying is clearly indicated as a potential problem among Nepali youth (72), we also acknowledge the existence of other socio-cultural environments in adolescent lives which are likely to also influence well-being outcomes. Adolescent health and development is strongly affected by social factors including family (73–75) and academic pressure/competition (76). Furthermore, there is also evidence of how social support can buffer against stressful situations (76–78). We control for each of these elements through FE^*^, AP^*^, and SS^*^, respectively. SS^*^ is measured based on six related variables, while the other latent construct have four each, in a manner consistent with prior work on adolescent well-being [such as (20, 79)]. Additional details on these constructs and all other variables, including descriptive statistics according to gender-grouping are presented in the Appendix in Supplementary Material, along with a correlation matrix of the key scale variables to provide preliminary insights on the hypothesized relationships.

## Estimation Strategy

While a reduced form system of equations may be a natural way to illustrate the model and causal channels laid out in Equations (1–3) above, doing so may mask underlying channels/complexities central to our narrative. The presence of three latent structures which play important controlling roles in our structural model requires a different estimation strategy allowing the flexibility to account for the measurement error associated with these constructs. Structural equation modeling (SEM) was chosen as the primary estimation strategy, given its application in prior work on health issues, family/peer dynamics, self-efficacy, depression, and psychotherapy (80).

Through maximum likelihood estimation, with iterative computation, SEM allows us to combine measurement models, which involve the relationships between observed (i.e., exogenous) measurements and latent constructs, with path analysis models that relate all variables. SEM allows one to model the measurement error (as opposed to the traditional statistical approach of assuming no error). SEM also eliminates problems with multicollinearity and reduces random error through utilizing multiple measures to describe one latent construct. Finally, use of SEM allows one to specify the appropriate variance-covariance structure for the system of equations based on prior knowledge and assumptions. In straight forward terms, the goal in SEM is to summarize this variance-covariance matrix and compare the estimated/implied variance-covariance matrix from the proposed structural model to the observed matrix from the data to explain as much of the variance as possible (81).

With these capabilities in mind, our structural model is enhanced with the addition of a measurement model representing the latent variables. Within each equation, *j* is the number of measurement variables associated with the respective latent construct.

(4) $$\begin{array}{l} AP_{ji}=\phi_{0}+\phi_{j1}AP_{i}^{*}+u_{4j}\;\forall \;j=1,\ldots ,4 \end{array}$$

(5) $$\begin{array}{l} FE_{ji}=\Upsilon_{0}+\Upsilon_{j1}FE_{i}^{*}+u_{5j}\;\forall \;j=1,\ldots ,4 \end{array}$$

(6) $$\begin{array}{l} SS_{ji}=\delta_{0}+\delta_{j1}SS_{i}^{*}+u_{6j}\;\forall \;j=1,\ldots ,6 \end{array}$$

We undertook two estimation methods of our full system: linear probability and generalized linear approaches. The key difference, as it pertains to our work, is the treatment of the measurement components for our latent constructs. Likert-scale survey items are often treated as interval; however, it is often more accurate to treat them as ordinal^2^. A generalized linear approach can be used under such scenarios where the estimation involves non-continuous variables which cannot be estimated under the typical assumptions of linear regression (81) ^3^. Under both approaches, we assume block-independence between the structural (Equations, 1-3) and the measurement (Equations, 4-6) systems. Additionally, we note that the parameter estimate of the respective latent variable's impact on the first measured indicator for each latent variable (e.g*., SS*_1*i*_*, FE*_1*i*_*, AP*_1*i*_) is normalized to 1 so that the magnitude of the latent constructs can be pegged against that measure.

With the potential for simultaneity between *PMPU* and the well-being outcomes (particularly *A)* implied by literature, and in the absence of over-identification testing typically associated with traditional regression techniques, we explored the strength of the model using three different covariance and model specification structures for the exogenous/observed variables. These results are presented in **Tables 3**, **4**. Our full model specified covariances be estimated between *Anx* and *Grit, PMPU* and *Anx*, and *PMPU* and *Grit*, with no demographic controls included in the equation for *PMPU*. The second structure constrained the covariance between *PMPU* and *Anx* and SS^*^ and FE^*^ to be zero, while maintaining no demographic controls in the equation for *PMPU*. The third model allowed for demographic controls in the *PMPU* equation. Goodness of fit diagnostics are reported for those results estimated under linear probability assumptions.

We determined the best fit model between the three models based on lowest Akaike's Information Criteria (AIC), and the remainder of analyses were performed based on this choice, for each estimation method. Assessment of the construct validity of the measurement models was performed, and order and rank conditions confirmed for model identification. Estimation of indirect and total effects for the various pressure points (most notably for *B)* was also performed, with results presented in **Tables 4**, **5**.

Various sensitivity analyses were performed to test the strength of our findings, including the use of traditional econometric techniques which are often conventionally employed to manage various forms of endogeneity including measurement error and simultaneity. Detailed information on such approaches and their results can be found in the Appendix in Supplementary Material.

## Results

### Basic Statistics

Summary statistics of all variables used are presented in Table 1. The study sample had an average age of 17.6, with 51% of them being female. Among the sample, 48% came from rural secondary schools. The average cost of mobile phones was 19.35 (thousand) NR.

The mean score of *Anx* was 13.38, which according to previous validation of the BAI (64) indicate at least mild levels of anxiety, on average, in the study sample^4^. *Grit* showed an average score of 3.26, and the average *PMPU* score was 86.65. This indicates more than half of the population have indicators of addictive mobile phone use, placing them above the midline of the scale. *FPMPU* had an average score of 20.09, indicating respondents perceive on average, even higher levels of problematic use among their closest friend than when responding to assessments of their own use.

There are overall very high indications of pressure in the academic environment to perform well in school, along with perceptions of the environment as being highly controlling and competitive (average scores around 4 on a 1/5 agreement scale). There is also a measurable presence of violence and strict oversight within the family/cultural environment, as indicated by average scores between 2.5 and 3.5 on a 1/5 agreement scale. With regard to social support measures, more than a quarter of those adolescents in the sample do not have someone from whom they could borrow money from or stay with in times of trouble. Further, more than one-third of the sample claim they have no one to confide in about violence or to deal with harassing situations, and less than half of the adolescents surveyed have a place to meet same sex friends or are a member of a social club/youth group.

### Estimation Results

In Table 2 are the results of estimating our full structural model with the latent variables measured according to linear probability estimation. Across all models, the fit statistics meet standard criterion/practices for assessing goodness of fit (81, 82). Parameter estimates indicate a statistically significant effect of *PMPU* on both well-being outcomes. Higher *PMPU* scores indicate lower *Grit* and higher *Anx. Bully* has a statistically significant impact on well-being measures, with the direction of association mirroring that of *PMPU*. There is no statistically significant effect of *PMPU* with *Anx*, but there is a positive covariance with *Grit*. There is a statistically significant, negative, covariance between the two well-being measures, across all models. These results are in-line with those indicated from preliminary Pearson correlations (found in the Appendix in Supplementary Material) indicating statistically significant inverse correlations between *PMPU* and both *Grit* and *Bully*, and a positive correlation between *PMPU* and *Anx*.

**Table 2:** Results of linear probability estimation for structural model of adolescent life influences on well-being.

	**(1)**			**(2)**			**(3)**		
	**Full model**			**Constraining non-sig. co-variances to zero**			**Constraining non-sig. co-variances to zero & control variables in PMPU Eq**.		
**Variables**	**Grit**	**Anxiety**	**PMPU score**	**Grit**	**Anxiety**	**PMPU score**	**Grit**	**Anxiety**	**PMPU score**
*PMPU*	−0.012^***^	0.077^*^		−0.012^***^	0.088^***^		−0.013^***^	0.088^***^	
	**(0.003)**	**(0.043)**		**(0.003)**	**(0.020)**		**(0.003)**	**(0.021)**	
*B*	−0.070^**^	1.506^***^	0.669	−0.070^**^	1.488^***^	0.668	−0.069^**^	1.493^***^	0.903
	**(0.030)**	**(0.477)**	(1.027)	**0.030**	**(0.471)**	(1.027)	**(0.030)**	**(0.472)**	(1.026)
AP^*^	0.177^**^	0.233	4.681^**^	0.178^**^	0.095	4.642^*^	0.194^**^	0.152	7.162^***^
	**(0.077)**	(1.241)	**(2.372)**	**0.078**	(1.131)	**(2.378)**	**(0.083)**	(1.142)	**(2.594)**
FE^*^*	−0.106	0.298	9.244^***^	−0.102	0.094	9.284^***^	−0.104	0.060	7.894^***^
	(0.073)	(1.144)	**(2.575)**	0.073	(1.010)	**(2.596)**	(0.072)	(0.994)	**(2.528)**
SS^*^	0.281	−3.416	−0.285	0.273	−3.401	0.256	0.283	−3.441	2.073
	(0.176)	(2.762)	(5.489)	0.175	(2.745)	(5.439)	(0.177)	(2.756)	(5.923)
*PC*			0.368^***^			0.363^***^			0.298^***^
			**(0.122)**			**(0.122)**			**(0.123)**
*PC2*			−0.003^**^			−0.003^**^			−0.003^**^
			**(0.001)**			**(0.001)**			**(0.001)**
*FPMPU*			1.693^***^			1.695^***^			1.601^***^
			**(0.159)**			**(0.159)**			**(0.165)**
*X1*	−0.070^*^	3.121^***^		−0.075^*^	3.136^***^		−0.089^**^	3.125^***^	−2.234
	**(0.0297)**	**(0.704)**		**0.043**	**(0.702)**		**(0.045)**	**(0.705)**	(1.525)
*X2*	0.010	0.424		0.010	0.417		0.005	0.416	−0.739
	(0.017)	(0.273)		0.017	(0.273)		(0.017)	(0.273)	(0.593)
*X3*	0.072	−1.561^**^		0.073	−1.532^**^		0.051	−1.547^**^	−3.855^**^
	(0.047)	**(0.744)**		0.047	**(0.755)**		(0.051)	**(0.759)**	**(1.663)**
*Constant*	4.19^***^	−2.116	48.06^***^	4.194^***^	−2.975	48.085^***^	4.327^***^	−2.937	66.765^***^
	**(0.374)**	(6.105)	**(3.651)**	**0.373**	(5.317)	**(3.652)**	**(0.419)**	(5.370)	**(11.280)**
Cov(Acad-Fam)	0.114^***^			0.111^***^			0.114^***^		
Cov(Acad-SS)	0.025^***^			0.024^***^			0.024^***^		
Cov(Fam-SS)	0.007			Constrained to Zero			Constrained to Zero		
Cov(BAI-Grit)	−0.337^**^			−0.353^**^			−0.355^**^		
Cov(BAI-PMPU)	3.121			Constrained to Zero			Constrained to Zero		
Cov(Grit-PMPU)		1.257^*^			1.281^*^			1.296^*^	
N	539			539			539		
ln(L)	−23330.248			−23330.723			−23326.214		
AIC	46856.497			46853.446			46850.428		
**Diagnostic tests**									
$\chi_{\text{ms}}^{2}$(dof,)	356.228(191); p> χ^2^ =0			357.177(193); p> χ^2^ =0			348.16 (190); p> χ^2^ =0		
CFI	0.883			0.884			0.888		
RMSEA	0.041			0.040			0.040		
SRMR	0.047			0.048			0.047		
CD	0.959			0.959			0.959		

*Standard errors in parentheses*;

*** *p < 0.01*,

** *p < 0.05*,

* *p < 0.1*.

*AIC, Akaike information criterion; CFI, Comparative Fit Index; RMSEA, Root Mean Square Error of Approximation; SRMR, Standardized Root Mean Square Residual; CD, Coefficient of Determination; $\chi_{\text{ms}}^{2}$, Chi-Sq. Test of the Likelihood Ratio Between the Specified and Saturated Model. Bolded values are those that are statistically significant*.

AP^*^ has a positive significant effect on *Grit* and *PMPU*, but no effect on *Anx*. FE^*^ appears to have a statistically significant positive effect on *PMPU*, only. Estimated covariances indicate significant positive associations between the latent variables representing the family and academic environments, and between the academic environment and social support.

All three measures used to allow for identification of the model (the “instruments”) and used to explain *PMPU*, are statistically significant. *PC* and *FPMPU h*ave a positive relationship with respondent's *PMPU* measure, and *PC2* a negative association. While results do not indicate any significant effects of demographic controls on *PMPU*, females (*X1)* are predicted to have lower grit and higher anxiety. Rural adolescents (*X3)* appear to have lower scores on our measure of anxiety, as well.

The results based on generalized linear modeling map similarly to those results presented above and are presented in Table 3. Again, *PMPU* and *Bully* are shown to have statistically significant relationships with well-being outcomes (positive for *Anx*, negative for *Grit*). Here, we see significant positive effects of *Bully* on *PMPU*, along with those previously seen from the latent pressures in the academic and family environments. Females and rural adolescent continue to be indicated to have positive and negative effects, respectively, on *Anx*, with no significant effects on *Grit*. Older adolescents, are also seen to have higher *A* under this approach. The measures used for identification and the referenced key covariances from above continue to be statistically significant as before.

**Table 3:** Results of generalized linear estimation for structural model of adolescent life influences on well-being.

	**(1) Full model**			**(2) Constraining non-sig. covariances to 0**			**(3) Constraining non-sig. covariances to 0 & control variables in PMPU Eq**.		
**Variables**	**Grit**	**Anxiety**	**PMPU score**	**Grit**	**Anxiety**	**PMPU score**	**Grit**	**Anxiety**	**PMPU score**
*PMPU*	−0.013^***^	0.099^***^		−0.013^***^	0.097^***^		−0.013^***^	0.097^***^	
	**(0.002)**	(0**.030)**		**(0.002)**	**(0.015)**		**(0.002)**	**(0.014)**	
*B*	−0.088^***^	1.892^***^	1.390^**^	−0.088^***^	1.898^***^	1.390^**^	−0.089^***^	1.896^***^	1.243^**^
	**(0.025)**	**(0.329)**	**(0.688)**	**(0.025)**	**(0.290)**	**(0.688)**	**(0.025)**	**(0.291)**	**(0.629)**
AP*	0.111^**^	–.403	1.519	0.111^**^	−0.393	1.518	0.115^**^	−0.379	2.391^**^
	**(0.050)**	(0.421)	(1.074)	**(0.050)**	(0.392)	(1.074)	**(0.049)**	(0.382)	**(1.089)**
FE*	−0.072	0.399	6.794^***^	−0.072	0.408	6.804^***^	−0.075	0.399	6.269^**^
	(0.050)	(0.504)	**(2.506)**	(0.050)	(0.506)	**(2.488)**	(0.050)	(0.502)	**(2.477)**
SS*	0.022	–.383	−0.215	0.022	−0.380	−0.191	0.022	−0.382	−0.154
	(0.030)	(0.317)	(0.879)	(0.030)	(0.318)	(0.887)	(0.030)	(0.317)	(0.861)
*PC*			0.270^***^			0.271^***^			0.276^***^
			**(0.093)**			**(0.091)**			**(0.098)**
*PC2*			−0.003^**^			−0.003^**^			−0.003^**^
			**(0.001)**			**(0.001)**			**(0.001)**
*FPMPU*			1.888^***^			1.888^***^			1.844^***^
			**(0.174)**			**(0.174)**			**(0.185)**
*X1*	−0.066	3.021^***^		−0.066	3.018^***^		−0.077	3.017^***^	−1.866
	(0.053)	**(0.789)**		(0.053)	**(0.782)**		(0.055)	**(0.782)**	(2.021)
*X2*	0.007	0.503^**^		0.007	0.505^**^		0.004	0.504^**^	−0.481
	(0.016)	(0**.223)**		(0.016)	**(0.228)**		(0.016)	**(0.228)**	(0.491)
*X3*	0.116^**^	−1.219^**^		0.116^**^	−1.220^**^		0.102^*^	−1.222^**^	−2.217
	**(0.054)**	(0**.505)**		**(0.054)**	**(0.500)**		**(0.053)**	**(0.500)**	(1.692)
*Constant*	4.263^***^	−5.819	44.519^***^	4.262^***^	−5.698	44.509^***^	4.336^***^	−5.715	56.011^***^
	**(0.369)**	(5.614)	**(3.546)**	**(0.370)**	(4.913)	**(3.552)**	**(0.372)**	(4.906)	**(8.479)**
Cov(Acad-Fam)	0.265^**^			0.262^**^			0.268^**^		
Cov(Acad-SS)	0.323^**^			0.317^***^			0.320^***^		
Cov(Fam-SS)	0.019			Constrained to Zero			Constrained to Zero		
Cov(BAI-Grit)	−0.297^***^			−0.293^***^			−0.295^***^		
Cov(BAI-PMPU)	−0.582			Constrained to Zero			Constrained to Zero		
Cov(Grit-PMPU)	1.533^***^			1.530^***^			1.502^***^		
N	539			539			539		
ln(L)	−11643.04			−11643.1			−11592.51		
AIC	23314.08			23314.2			23211.02		

*Standard errors in parentheses*;

*** *p < 0.01*,

** *p < 0.05*,

* *p < 0.1*;

*AIC, Akaike information criterion. Bolded values are those that are statistically significant*.

Based on AIC values, the results presented in column (3) of both Tables 3, 4, indicate the best fit between the three model structures. Thus, all subsequent analyses were based on this approach. As previously indicated, full assessment of a structural model also requires assessment of the validity of the constructs used in the measurement model portion of the full model. Average variance extracted (AVE) and construct reliability (CR) values for our model are all above the 0.7 level indicated for good convergent validity (81). To assess discriminant validity, the AVE value should be larger than the squared correlations between two constructs, to reduce any lingering multicollinearity issues (81). For our model, this does not appear to be the case, regardless of estimation approach. Thus, the measurement model was also deemed to have divergent validity.

**Table 4:** Mediation analysis for linear probability estimation of structural model of adolescent life influences on well-being.

**Treatment variable**	**Indirect effect (IE)**	**Direct effect (DE)**	**Total effect (TE)**
**PANEL A: Anxiety**			
**Mediator variable: PMPU score**			
*B*	0.079 (0.092)	1.493^***^(0.472)	1.572^***^ **(0.479)**
AP^*^	0.631^**^ **(0.269)**	0.152(1.142)	0.783 (1.123)
FE^*^	0.695^***^ **(0.279)**	0.060(0.994)	0.847 (1.103)
SS^*^	0.182 (0.526)	−3.441(2.756)	−3.259 (2.802)
*X1*	−0.197 (0.163)	3.125^***^(**0.705**)	2.928^***^ (**0.713)**
*X2*	−0.065 (0.054)	0.416(0.273)	0.351 (0.278)
*X3*	−0.339^**^ **(0.168)**	−1.547^**^(**0.759**)	−1.887^**^ **(0.761)**
**PANEL B: Grit**			
**Mediator variable** **=** **PMPU score**			
*B*	−0.011 (0.013)	−0.069^**^(**0.030**)	−0.080^***^ **(0.029)**
AP^*^	−0.091^**^ **(0.040)**	0.194^**^(**0.083**)	0.103 (0.072)
FE*	−0.100^**^ **(0.039)**	−0.104(0.072)	0.094 (0.082)
SS*	−0.026 (0.075)	0.283(0.177)	0.257 (0.177)
*X1*	0.028 (0.021)	−0.089^**^(**0.045**)	−0.060 (0.044)
*X2*	0.009 (0.008)	0.005(0.017)	0.015 (0.017)
*X3*	0.049^**^ **(0.024)**	0.051(0.051)	0.055 (0.056)
**PANEL C: Mediation pathway diagram**			
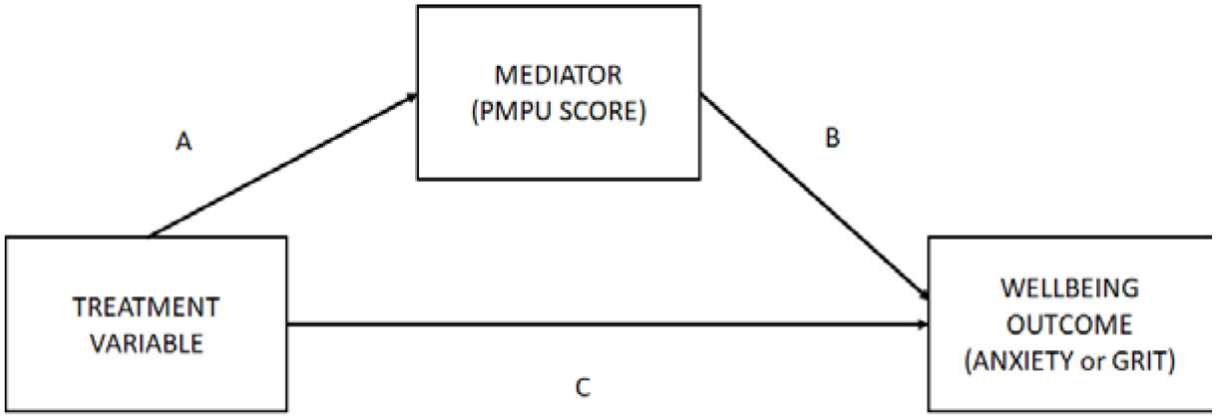			

*Standard errors (obtained by the delta method) in parentheses*;

*** *p < 0.01*,

** *p < 0.05*,

* *p < 0.1*.

*Bolded values are those that are statistically significant*.

**Figure 3:**
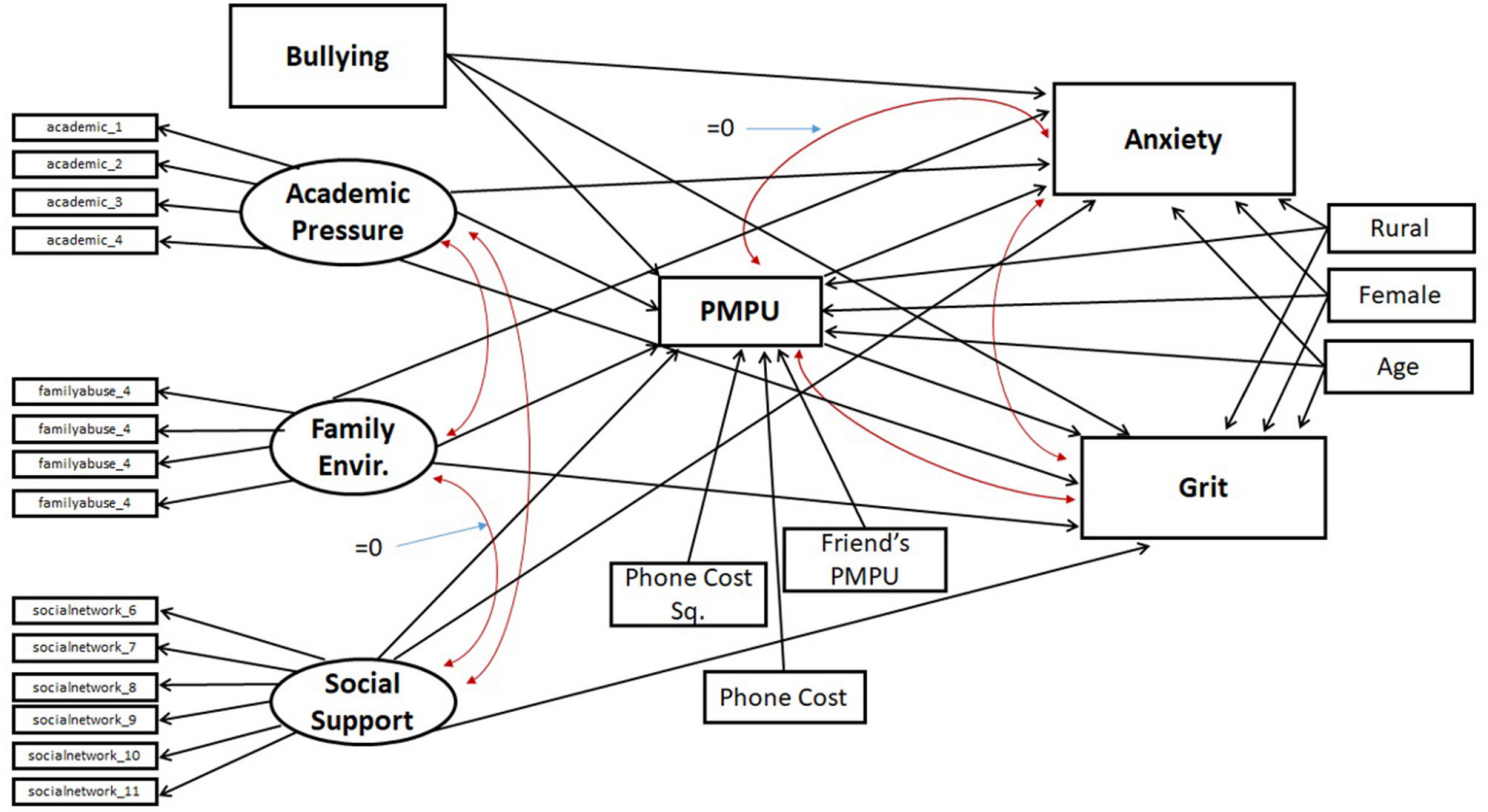
Final structural model.

Mediation analysis entails disaggregating the indirect and direct effects of the mediation pathway depicted in Panel C of Table 5. As visualized in the full structural model (approach number three) depicted in Figure 3, the treatment variables analyzed are those seven variables which have the potential to exhibit an indirect effect (e.g., they have a pathway which passes through *PMPU*).

**Table 5:** Mediation analysis for generalized linear estimation of structural model of adolescent life influences on well-being.

**Treatment variable**	**Indirect effect (IE)**	**Direct effect (DE)**	**Total effect (TE)**
**PANEL A: Anxiety**			
**Mediator variable: PMPU score**			
*B*	0.121^*^ **(0.068)**	1.896^***^ (**0.291**)	2.018^***^ **(0.302)**
AP^*^	0.233^**^ **(0.110)**	−0.379(0.382)	−0.146 (0.392)
FE^*^	0.611^***^ **(0.216)**	0.399(0.502)	0.232 (0.455)
SS^*^	−0.015 (0.084)	−0.382(0.317)	−0.397 (0.312)
*X1*	−0.182 (0.209)	3.017^***^(**0.782**)	2.835^***^ **(0.866)**
*X2*	−0.047 (0.052)	0.504^**^(0.228)	0.457^**^ **(0.218)**
*X3*	−0.216 (0.152)	−1.222^**^(**0.500**)	−1.438^***^ **(0.515)**
**PANEL B: Grit**			
**Mediator variable** **=** **PMPU score**			
*B*	−0.016^*^ **(0.009)**	−0.089^***^(**0.025**)	−0.105^***^ **(0.020)**
AP^*^	−0.031^*^ **(0.016)**	0.115^**^(**0.049**)	0.084^*^ **(0.047)**
FE^*^	−0.080^**^ **(0.033)**	−0.075(0.050)	0.035 (0.042)
SS^*^	0.002 (0.011)	0.022(0.030)	0.024 (0.034)
*X1*	0.024 (0.028)	−0.077(0.055)	−0.054 (0.056)
*X2*	0.006 (0.006)	0.004(0.016)	0.010 (0.017)
*X3*	0.028 (0.019)	0.102^*^(**0.053**)	0.112^*^ **(0.059)**
**PANEL C: Mediation pathway diagram**			
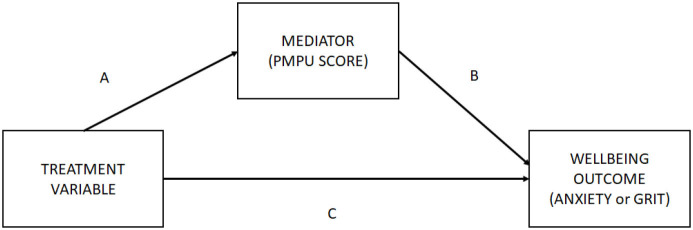			

*Standard errors (obtained by the delta method) in parentheses*;

*** *p < 0.01*,

** *p < 0.05*,

* *p < 0.1*.

*Bolded values are those that are statistically significant*.

Looking at Panels A and B in Tables 4, 5, there are consistently significant direct and total effects of *Bully* on both well-being outcome measures, regardless of estimation approach. Additionally, there are consistently significant direct and total effects of being female on anxiety. Interpretation of indirect and mediation effects is slightly different between the two estimation approaches. So, for the sake of brevity, we detail the results from generalized linear modeling, given our belief that the assumptions underlying the measurement model with this approach may more accurately reflect the underlying processes and relationships our structural model attempts to capture, based on the data generating processes.

Referring to Table 5, one can see significant indirect effects of Bully, AP^*^, and FE^*^ on both measures of well-being. This implies that there is a statistically significant impact on the well-being measures, which passes through the mediating variable of PMPU. FE^*^ only has a significant indirect effect (indicative of full mediation), whereas *Bully* has significant indirect and direct effects, indicating only partial mediation. As it pertains to impacts on Grit, AP^*^ appears to only be partially mediated, given significant indirect and direct effects. Interpretation of the mediation effects of *PMPU* on the relationship between AP^*^ and *Anx* is less clear. Overall, there are no mediation effects of *PMPU* on the relationships between demographic controls and well-being outcome measures.

## Discussion, Policy Implications and Conclusions

It is imperative that government and policy makers focus on ways to improve the well-being of adolescents, given current negative trajectories seen worldwide, and particularly in developing world nations such as Nepal. There is growing interest in using mobile-based (e.g., mHealth) interventions to combat such issues as high suicide and bullying rates (4). While adolescent use of mobile technology is believed to be high, existing literature focusing on mHealth has primarily focused on the believed cost and reach benefits potentially offered. To date, though, there has been a noted lack of economic data to support the use of digital health interventions (9), and coupled with that is a recognition of the importance of acknowledging potential unintended downsides to its implementation.

There is evidence from developed-world contexts of the negative mental health and well-being outcomes that are highly linked with overuse of mobile/smartphones (83, 84). Such findings are also being confirmed with initial studies of such issues in developing world contexts. However, clarity of the directionality of these relationships within the “dark side” of technology are proving hard to determine (28, 85). The small number of rigorous empirical examinations on these topics primarily neglect any underlying motivational framework to answer why adolescents are turning to their mobile phones. Or, if a motivational framework is emphasized, there is lack of strong statistical analyses to ensure validity of the theories postulated.

Thus, the overarching purpose of this study was to evaluate the presence and associations of PMPU in Nepali adolescents, using a strong analytic approach, framed by a well-supported conceptual theory. By incorporating assessment of both a positive and negative well-being outcome, we have attempted to capture a more holistic vision of the relationships underlying the connections between need thwarting contexts, PMPU, and well-being. We capture two measures of the potential for life success based on the downward trajectory chronic anxiety can provide, vs. the upward trajectory of grit. Using a full structural modeling approach, which accounts for the measurement error in socio-cultural environmental latent constructs and the covariance between key variables, we have been able to produce robust results which provide new insights and directions for future research into these topics.

As a consequence of this research, we have expanded mHealth literature by looking at the potential downsides of phone use which may come up as negative externalities to mHealth initiatives. In addition, by framing our analysis according to BPNT, we are able to capture motivational factors which may trigger turning to phones in the first place, and have moved beyond analyses based solely on personality traits [such as (86, 87)]^5^. Our application of BPNT has also moved that literature forward by applying it to technology use outside of video games/dating apps, and beyond applications in the classroom/academic functioning. We have also directly addressed simultaneity concerns in both our main modeling framework through accounting for covariances, and in our detailed sensitivity analyses using multiple econometric techniques and testing (summarized in Appendix in Supplementary Material).

Key results from our work indicate support for all four of our initial hypotheses. Those adolescents scoring higher on our measure of bullying are seen to exhibit higher levels of anxiety symptomology and lower on indicators of grit (H1). Those adolescent who report more bullying are seen to also have higher indicators for PMPU (H2). Beyond our hypotheses, literature which emphasizes the large portion of adolescents' time endowment spent at school (76, 91) allows for the significant direct effects of academic pressure on well-being outcomes to make sense. Similarly, the significant direct effects we see for females having higher anxiety measures is in congruence with existing research which indicates that negative mental health outcomes are often found to be more common among female (vs. male) adolescent populations (92, 93).

Significant indirect effects of bullying on well-being outcomes, mediated by our measure of PMPU, provide support for H3, and are the strongest indicators in support of BPNT being an appropriate framework through which to view the PMPU-well-being relationship. The additional significant indirect effects for pressure from the family environment and academic environment on well-being, mediated by PMPU, further provide support for the role that need thwarting contexts play in reducing overall well-being. These findings are in line with researchers who have pushed forward a “need-density” hypothesis, wherein those who have less basic needs met, may also be less able to autonomously regulate activities and make congruent decisions that match their aims and values (94, 95). They are therefore more vulnerable to technology overuse, and a vicious cycle can continue where seeking need fulfillment may ultimately further need frustration.

Our finding of a statistically significant covariance between our two outcomes measures, supports our final hypothesis (H4). Calculated as a negative correlation of −0.09, this finding supports our use of a full structural equation modeling approach which accounts for this interconnectedness. The ability to account for covariances between *PMPU* and the two well-being outcomes has also allowed us to realize a key insight into the lack of a significant covariance (i.e., simultaneous relationship) between *A* and *PMPU*. This finding appears contrary to much of the literature examining problematic phone use and mental health, which has pushed for understanding the relationship as bi-directional (28, 34). However, it is supported by findings by Pivetta et al. (96) who also did not find evidence of pre-existing psychopathology exerting a significant influence on average smartphone user's behavior. The stability of this finding is confirmed in our sensitivity analyses (see Appendix in Supplementary Material), where econometric tests for statistical exogeneity between our measures of PMPU and anxiety are not rejected.

Intriguingly, there are unexpected significant covariances (and concerns for endogeneity) indicated between PMPU and our measure of grit. Such a relationship has not been previously found or analyzed in work looking at mobile technology. Prior evidence of grit's influence on other maladaptive behaviors such as substance use (97, 98) and video game addiction (99) has been indicated in literature, but there is also literature which found no such associations (100, 101). The evidence supporting the important role that grit plays in the healthy development and success of individuals, and particularly adolescents is growing (102, 103). The connections between technology use and grit may offer a new and unique direction in which future research in these areas could focus.

In study of our mediation effects, there are instances in which indirect and/or direct effects are statistically significant, while total effects are not. It has been shown that it is possible to have such a situation, and this can be explained by the presence of several mediating paths that cancel each other out (104). This would be viewed as competitive mediation, as compared to complementary mediation where indirect and direct effects both exist and move in the same direction, or indirect-only mediation (105, 106). Should further exploration of each adolescent pressure/environmental factor accounted for in our model be pursued, such work may entail further breaking down analyses of such pathways.

Future work may also benefit from further exploration of additional unexpected outcomes in this study, including the lack of significance of SS^*^. While not part of our research hypotheses, the findings of complete lack of significance in our main estimation models for this variable is contrary to what is found in typical regression approaches to the system of equations we estimate (see Appendix in Supplementary Material), and what would be expected from literature (77, 107). Initial speculation for this outcome revolves around the possibility that this was a latent construct. AVE and CR values are the lowest for social support, which may indicate that the strength of our measures for this construct are weaker than the other two.

Additional limitations to our work include that while many studies listed previously have looked at impacts of smartphone use on adolescents' sleeping problems, we did not specifically measure this or focus on it, although there are references to sleep troubles within the PMPU survey instrument we used. We also could have used a different set of explanatory “instrumental variables” to ensure identification within our equation for *PMPU*. Previous authors who have used instrumental variable techniques similar to those used in our sensitivity analyses have captured phone use (not necessarily problematic use phone) with measures of Wi-Fi capabilities, phone download volume, and 4G streaming capabilities (69, 108). The limitations in internet coverage in the developing world and length limitations in our survey to avoid survey fatigue meant that we were limited on which elements of adolescents' technology use to capture. However, future empirical work could consider capturing such measures if additional survey development on this topic were to be pursued. In any future survey development on these topics, it might also be useful to consider inclusion of a specific need thwarting/need satisfaction scale (109) to delineate differences in the three needs of autonomy, relatedness, and competence and their unique roles in the relationships indicated by our model.

By incorporating key specific factors of life adolescents face which can satisfy/thwart needs in this study, and viewing need thwarting through a more all-encompassing lens [like (56, 110)], we are able to gain insights into ways that future interventions can be focused. Additionally, results of this study are confirmation of the universality of needs (111, 112), and additional confirmation that BPNT applies across cultures. This implies that the best way to focus any health/well-being interventions aimed at Nepali adolescents would be to seek out ways to improve their perceptions that their needs for autonomy, relatedness, and competence are being met.

Our results do support literature's findings of poorer well-being outcomes from maladaptive/problematic use of mobile phones. Consequently, care must be taken if mHealth interventions are the path that policymakers choose, given that many mental health apps have not been properly vetted/tested (113, 114). If such interventions are deployed in conjunction with, or subsequent to, non-technology based interventions which focus on meeting the three basic needs, such concerns may be dampened. If adolescents no longer see mobile phones as a compensatory means by which to fulfill their unmet or thwarted needs in life, then the prevalence of problematic phone use may go down, and any negative well-being outcomes from that maladaptive use would also fall.

Work in the developed world has focused on the roles of in-school health systems and counselors as a potential front-line approach to negative well-being outcomes (115, 116). However, in many developing world contexts, there is not even a school nurse, let alone a mental/emotional counselor. Among our sample, 62.4% report that there is a school counselor at their school, while 85.2% claim that they would like one present with whom they could discuss their worries/concerns. So, a first step in non-technology-based interventions would be to try to raise the perceived importance of having health professionals on staff at schools and reducing associated stigma. However, in the absence of financial support for such initiatives, or as the recent global pandemic which removed in-person schooling opportunities across the globe have shown, there may be times where society must rely on digital approaches to address the socio-emotional needs of youth (117, 118). It is in the face of such situations, that careful consideration must be given to proper form and approach in digitally-based approaches to healthcare, including mental/emotional support.

If mHealth interventions/approaches are properly focused, some studies have shown both passive and active entertainment media, including social media, can provide challenges, choices, and relational elements potentially conducive to need satisfaction (119). Apps such as the “Woebot” (120) and the “Youper” (121) have being deployed which, unlike traditional mHealth apps that focus on mind-calming exercises or simply providing resources, utilize an AI chatbot which chats with you like a friend, asking questions, and assessing your responses. This type of application genuinely may fulfill such needs as relatedness and autonomy.

Additionally, there needs to be buy-in and input from the end-users in the platform/app development, to ensure the highest likelihood and levels of engagement which match the needs and desires of the end-user patients (122, 123). It may be useful to create gender-targeted interventions, given evidence from literature and supported in this work that females exhibit higher anxiety symptomology (124). Specifically in the Nepali context, females face strong pressures for early marriage (19, 125) and menstrual taboos (126) which may exacerbate any lack of need fulfillment. Furthermore, in Nepal it is common for adolescents to share their phone with one or more other individuals. Consequently, privacy concerns may be a real barrier to full adoption/success of mobile-based interventions. Within our study, over 30% of adolescents report that they are not the only user of their phone. Close to 32% of the adolescents surveyed say that they worry about their privacy, consistent with 53% of them reporting that others take and go through their phones without permission.

Overall, though, investing in health systems and looking at minimizing the treatment gap is forecast to produce economic returns of 2.3 to 3.0 to 1 by 2030, indicating a long-term gain of $2.3–3.00 for every $1 invested in prevention and treatment (127). Such figures are based on not only reduction in long-term healthcare costs, but also increased economic productivity of healthier individuals contributing to society. In less than 10 years, today's adolescent populations will be those contributors to society. The “unhappiness” Inverted-U curve is real and ubiquitous across the world (128, 129), and if adolescents are already struggling, they are only going to get that much worse down the road. By finding ways to improve their positive well-being, now, there are enormous gains to be had long-term. Our work has shown that while PMPU does indeed appear to be a culprit of some of the negative well-being outcomes seen in a developing-world context such as Nepal, this connection can also be partially explained by the preexisting need thwarting contexts that these adolescent face. Contexts that drive them to phones in an attempt to meet their needs. Finding ways to meet those needs, whether through carefully targeted mHealth apps or non-technology-based approaches, may be a critical step toward having a next generation of world leaders who surpass the current in their levels of personal, emotional, and financial success.

## Data Availability Statement

The raw data supporting the conclusions of this article will be made available by the authors, without undue reservation.

## Ethics Statement

The studies involving human participants were reviewed and approved by Institutional Review Board at the University of New Mexico. Written informed consent to participate in this study was provided by the participants' legal guardian/next of kin.

## Author Contributions

SY researched all background literatures, conceived of the primary research questions, generated the survey, led the IRB approval process, performed data analysis, drafted and edited the article. AB contributed to survey development, article formatting and editing, analysis guidance, and approval of all contents as appropriate to be listed as an author.

## Conflict of Interest

The authors declare that the research was conducted in the absence of any commercial or financial relationships that could be construed as a potential conflict of interest.

## Footnotes

^1^Anxiety is a measure of feelings of worry, nervousness, or unease, typically about an imminent event or something with an uncertain outcome, and is a typical measure of negative well-being, which hinders a multitude of long-term successes (48–50). Grit, on the other hand, is a measure of perseverance and passion for achieving long-term goals, which serves as a useful measure of positive well-being potential and ability to succeed in life. Grittier people have been shown to have greater educational attainment/achievement, entrepreneurial potential, and earnings (51, 52).

^2^The difference from an answer of “Strongly Agree” to “Agree” (coded as 1 and 2) is not treated as equivalent to that of between “Agree” and “Neutral” (coded as 2 and 3) in an intuitive sense under an ordinal treatment.

^3^Through SEM based on a generalized linear model, the estimation is essentially a generalization of nonlinear least squares, where the likelihood is derived under the assumption that each observed variable is independent and identically distributed (*iid*) across the estimation sample and observed variables are also assumed to be independent of each other. These assumptions are conditional on the latent variables and observed exogenous variables. This is opposed to estimation with a linear probability treatment of the observed measures, where the likelihood is derived based on the means, variances, and covariances of the observed exogenous variables (not conditional upon). Additionally, estimation based on the generalized linear model removes any assumptions of normality of the observed exogenous variables, and instead uses a link function such as logit for distributional families such as ordinal or Bernoulli.

^4^Note that use of this survey instrument was not meant to diagnose generalized anxiety disorder, but rather to serve as an indicator of overall experience of anxiety-related symptoms – indicative of negatively oriented emotional and mental well-being.

^5^Unlike typical personality traits such as neuroticisms or introversion, grit has been viewed and confirmed as a learned characteristic, which develops over time and can be affected by life experiences (88, 89), much like anxiety has traditionally been recognized as situation/environmentally triggered/dependent (90).

## References

[1] UNICEF. Adolescents Overview. Unicef Data (2019). Available online at: https://data.unicef.org/topic/adolescents/overview/. (accessed March 1, 2020).

[2] UN. Report of the United Nations Expert Group Meeting on Adolescents, Youth and Development. New York, NY (2011).

[3] Patalay P Fitzsimons E. Development and predictors of mental ill-health and wellbeing from childhood to adolescence. Soc Psychiatry Psychiatr Epidemiol. (2018) 53:1311–23. 10.1007/s00127-018-1604-0 30259056

[4] WHO-SEARO. Mental Health Status of Adolescents in South-East Asia: Evidence for Action. New Delhi (2017). Available online at: http://www.searo.who.int/entity/mental_health/documents/9789290225737/en/. (accessed March 1, 2020).

[5] Souraya S Canning T Farmer P. Addressing Anxiety and Depression: A Whole System Approach. Doha: WISH Anxiety and Depression Forum 2018 (2018). Available online at: https://www.wish.org.qa/wp-content/uploads/2018/11/IMPJ6078-WISH-2018-Anxiety-and-Depression-181026.pdf. (accessed March 1, 2020).

[6] Naslund JA Aschbrenner KA Araya R Marsch LA Unützer J Patel V. Digital technology for treating and preventing mental disorders in low-income and middle-income countries: a narrative review of the literature. Lancet Psychiatry. (2017) 4:486–500. 10.1016/S2215-0366(17)30096-2 28433615 PMC5523650

[7] Payne HE Lister C West JH Bernhardt JM. Behavioral functionality of mobile apps in health interventions: a systematic review of the literature. JMIR MHealth UHealth. (2015) 3:e20. 10.2196/mhealth.3335 25803705 PMC4376122

[8] Zhao J Freeman B Li M. Can mobile phone apps influence people's health behavior change? An evidence review. J Med Intern Res. (2016) 18:e287. 10.2196/jmir.5692 27806926 PMC5295827

[9] Iribarren SJ Cato K Falzon L Stone PW. What is the economic evidence for MHealth? A systematic review of economic evaluations of mhealth solutions edited by cathy mihalopoulos. PLoS ONE. (2017) 12:e0170581. 10.1371/journal.pone.0170581 28152012 PMC5289471

[10] Pew Research Center. Teens, Social Media & Technology. Report (2018).

[11] Rideout V Robb MB. The Common Sense Census: Media Use by Tweens and Teens, 2019. San Francisco, CA: Common Sense Media (2019).

[12] Radovic A Badawy SM. Technology use for adolescent health and wellness. Pediatrics. (2020) 145(Suppl. 2):S186–94. 10.1542/peds.2019-2056G 32358210

[13] Grist R Porter J Stallard P. Mental health mobile apps for preadolescents and adolescents: a systematic review. J Med Intern Res. (2017) 19:e176. 10.2196/jmir.7332 28546138 PMC5465380

[14] Hollis C Falconer CJ Martin JL Whittington C Stockton S Glazebrook C. Annual research review: digital health interventions for children and young people with mental health problems - a systematic and meta-review. J Child Psychol Psychiatry. (2017) 58:474–503. 10.1111/jcpp.12663 27943285

[15] Sohn S Rees P Wildridge B Kalk NJ Carter B. Prevalence of problematic smartphone usage and associated mental health outcomes amongst children and young people: a systematic review, meta-analysis and GRADE of the evidence. BMC Psychiatry. (2019) 19:356. 10.1186/s12888-019-2393-z 31779637 PMC6883663

[16] Stiglic N Viner RM. Effects of screentime on the health and well-being of children and adolescents: a systematic review of reviews. BMJ Open. (2019) 9:e023191. 10.1136/bmjopen-2018-023191 30606703 PMC6326346

[17] Davey S Davey A. Assessment of smartphone addiction in Indian adolescents: a mixed method study by systematic-review and meta-analysis approach. Int J Prev Med. (2014) 5:1500–11. 25709785 PMC4336980

[18] Patel SASAW Puri PA. A Study of mobile phone addiction and mental health among adolescent girls studying in various streams. Int J Indian Psychol. (2017) 5:146–51. 10.25215/0501.099

[19] Adhikari RP Upadhaya N Pokharel R Suwal BR Shrestha MP Subedi PK. Health and social vulnerability of adolescents in Nepal. SM J Public Health Epidemiol. (2016) 2:1032.

[20] Amin S Bajracharya A Chau M Puri M. Highlights from the Unicef Adolescent Development and Participation Baseline Study. New York, NY: Population Council (2014). 10.31899/pgy10.1017

[21] Ghimire A Samuels F Giri I Adhikari P. Communication Strategies for Addressing Discriminatory Social Norms in Marriage and Education for Adolescent Girls in Nepal. London: Overseas Development Institute (ODI) (2015).

[22] UNDP editor. Human Development for Everyone. Human Development Report 2016. New York, NY: United Nations Development Programme (2016).

[23] World Bank. The Little Data Book on Information and Communication Technology 2017. The World Bank (2017).

[24] Kamrudin A. Assessing the mental health of girls in Nepal: a study of the impact of societal factors on former child soldiers conscripted into Nepal's maoist movement and non-conscripted children Atlanta, GA: Emory University (2009).

[25] Luitel NP Jordans MJD Sapkota RP Tol WA Kohrt BA Thapa SB. Conflict and mental health: a cross-sectional epidemiological study in Nepal. Soc Psychiatry Psychiatr Epidemiol. (2013) 48:183–93. 10.1007/s00127-012-0539-0 22777395

[26] Meyers J Ozonoff A Baruwal A Pande S Harsha A Sharma R. Combining healthcare-based and participatory approaches to surveillance: trends in diarrheal and respiratory conditions collected by a mobile phone system by community health workers in rural Nepal. Edited by Delmiro Fernandez-Reyes. PLoS ONE 11: e0152738. 10.1371/journal.pone.0152738 PMC4844116 27111734

[27] Oh DH Dabbagh A Goodson JL Strebel PM Thapa S Giri JN. Real-Time Monitoring of Vaccination Campaign Performance Using Mobile Phones — Nepal, 2016. Vol. 65, No. 39. Morbidity and Mortality Weekly Report. US Department of Health Services/Centers for Disease Control and Prevention (2016). 10.15585/mmwr.mm6539a5 27711034

[28] Elhai JD Dvorak RD Levine JC Brian Hall J. Problematic smartphone use: a conceptual overview and systematic review of relations with anxiety and depression psychopathology. J Affect Disord. (2017) 207:251–59. 10.1016/j.jad.2016.08.030 27736736

[29] Reid Chassiakos Y Radesky J Christakis D Moreno MA Cross C Council On Communications and Media. Children and adolescents and digital media. Pediatrics. (2016) 138:e20162593. 10.1542/peds.2016-2593 27940795

[30] Vernon L Modecki KL Barber BL. Mobile Phones in the bedroom: trajectories of sleep habits and subsequent adolescent psychosocial development. Child Dev. (2018) 89:66–77. 10.1111/cdev.12836 28556891

[31] Lemola S Perkinson-Gloor N Brand S Dewald-Kaufmann JF Grob A. Adolescents' electronic media use at night, sleep disturbance, and depressive symptoms in the smartphone age. J. Youth Adolesc. (2015) 44:405–18. 10.1007/s10964-014-0176-x 25204836

[32] Seo DG Park Y Kim MK Park J. Mobile phone dependency and its impacts on adolescents' social and academic behaviors. Comput Hum Behav. (2016) 63:282–92. 10.1016/j.chb.2016.05.026

[33] Harwood J Dooley JJ Scott AJ Joiner R. Constantly connected – The effects of smart-devices on mental health. Comput Hum Behav. (2014) 34:267–72. 10.1016/j.chb.2014.02.006

[34] Billieux J Van der Linden M Rochat L. The role of impulsivity in actual and problematic use of the mobile phone. Appl Cogn Psychol. (2008) 22:1195–210. 10.1002/acp.1429

[35] Cheever NA Rosen LD Carrier LM Chavez A. Out of sight is not out of mind: the impact of restricting wireless mobile device use on anxiety levels among low, moderate and high users. Comput Hum Behav. (2014) 37:290–97. 10.1016/j.chb.2014.05.002

[36] Clayton RB Leshner G Almond A. The extended iself: the impact of iphone separation on cognition, emotion, and physiology. J Comput Mediat Commun. (2015) 20:119–35. 10.1111/jcc4.12109

[37] Lu X Katoh T Chen Z Nagata T Kitamura T. Text messaging: are dependency and excessive use discretely different for Japanese University students? Psychiatry Res. (2014) 216:255–62. 10.1016/j.psychres.2013.12.024 24560613

[38] Jun S. The reciprocal longitudinal relationships between mobile phone addiction and depressive symptoms among korean adolescents. Comput Hum Behav. (2016) 58:179–86. 10.1016/j.chb.2015.12.061

[39] Eijnden RJJM Meerkerk GJA Vermulst A Spijkerman R Engels RCME. Online communication, compulsive internet use, and psychosocial well-being among adolescents: a longitudinal study. Dev Psychol. (2008) 44:655–65. 10.1037/0012-1649.44.3.655 18473634

[40] Yen JY Cheng-Fang Y Chen CS Chang YH Yeh YC Ko CH. The bidirectional interactions between addiction, behaviour approach and behaviour inhibition systems among adolescents in a prospective study. Psychiatry Res. (2012) 200:588–92. 10.1016/j.psychres.2012.03.015 22534501

[41] Ryan RM Deci EL. Self-determination theory and the facilitation of intrinsic motivation, social development, and well-being. Am Psychol. (2000) 55:68–78. 10.1037/0003-066X.55.1.68 11392867

[42] Zhang J Liu Y Zhang J Chen C. Linking psychological strain and suicide ideation: a test of the mediating effect of self-determination among Chinese workers. J Nerv Ment Dis. (2018) 206:362–9. 10.1097/NMD.0000000000000809 29570485

[43] Gui J Kono S Walker GJ. Basic psychological need satisfaction and affect within the leisure sphere. Leisure Stud. (2019) 38:114–27. 10.1080/02614367.2018.1539866

[44] Vandenkerckhove B Brenning K Vansteenkiste M Luyten P Soenens B. The explanatory role of basic psychological need experiences in the relation between dependency, self-criticism and psychopathology in adolescence. J Psychopathol Behav Assess. (2019) 41:574–88. 10.1007/s10862-019-09719-0

[45] Deci EL Vansteenkiste M. Self-determination theory and basic need satisfaction: understanding human development in positive psychology. Ricerche Psicol. (2004) 27:23–40.

[46] Masur PK Reinecke L Ziegele M Quiring O. The interplay of intrinsic need satisfaction and facebook specific motives in explaining addictive behavior on facebook. Comput Hum Behav. (2014) 39:376–86. 10.1016/j.chb.2014.05.047

[47] Young KS Yue XD Ying L. Prevalence estimates and etiologic models of internet addiction, In: Young KS de Abreu CN editors. Internet Addiction: A Handbook and Guide to Evaluation and Treatment Hoboken, NJ: John Wiley & Sons (2011). p. 3–17. 10.1002/9781118013991.ch1

[48] Knapp M Ardino V Brimblecombe N Evans-Lacko S Ieemi V King D. Youth Mental Health: New Economic Evidence. PSSRU: University of Kent. (2016). Available online at: https://www.pssru.ac.uk/publications/pub-5160/. (accessed March 1, 2020).

[49] Knapp M King D Healey A Thomas C. Economic outcomes in adulthood and their associations with antisocial conduct, attention deficit and anxiety problems in childhood. J Ment Health Policy Econ. (2011) 14:137–47. 22116171

[50] Snell T Knapp M Healey A Guglani S Evans-Lacko S Fernandez JL. Economic impact of childhood psychiatric disorder on public sector services in Britain: estimates from national survey data. J Child Psychol Psychiatry. (2013) 54:977–85. 10.1111/jcpp.12055 23442096

[51] Beyhan O. University students grit level and grit achievement relation. Soc Sci Educ Res Rev. (2016) 3:13–23.

[52] Mooradian T Matzler K Uzelac B Bauer F. Perspiration and inspiration: grit and innovativeness as antecedents of entrepreneurial success. J Econ Psychol. (2016) 56:232–43. 10.1016/j.joep.2016.08.001

[53] Shapka JD Onditi HZ Collie RJ Lapidot-Lefler N. Cyberbullying and cybervictimization within a cross-cultural context: a study of Canadian and Tanzanian adolescents. Child Dev. (2018) 89:89–99. 10.1111/cdev.12829 28523643

[54] McKinnon B Gariépy G Sentenac M Elgar FJ. Adolescent suicidal behaviours in 32 low- and middle-income countries. Bull World Health Organ. (2016) 94:340–50F. 10.2471/BLT.15.163295 27147764 PMC4850530

[55] Tian L Tian Q Huebner ES. School-related social support and adolescents' school-related subjective well-being: the mediating role of basic psychological needs satisfaction at school. Soc Indic Res. (2016) 128:105–29. 10.1007/s11205-015-1021-7

[56] Orkibi H Ronen T. Basic psychological needs satisfaction mediates the association between self-control skills and subjective well-being. Front Psychol. (2017) 8:936. 10.3389/fpsyg.2017.00936 28638362 PMC5461363

[57] Sheldon KM Gunz A. Psychological needs as basic motives, not just experiential requirements. J Pers. (2009) 77:1467–92. 10.1111/j.1467-6494.2009.00589.x 19678877

[58] Rigby CS Ryan RM. Chapter 3: Time Well Spent?. In: Reinecke L Oliver MB, editors. The Routledge Handbook of Media Use and Well-Being: International Perspectives on Theory and Research on Positive Media Effects. New York, NY: Routledge (2017). p. 34–48.

[59] Jin B Kim J. Grit basic needs satisfaction, and subjective well-being. J Individ Diff. (2017) 38:29. 10.1027/1614-0001/a000219

[60] Jiang W Jiang J Du X Gu D Sun Y Zhang Y. Striving and happiness: between- and within-person-level associations among grit, needs satisfaction and subjective well-being. J Posit Psychol. (2019) 15:543–55. 10.1080/17439760.2019.1639796

[61] Musumari PM Tangmunkongvorakul A Srithanaviboonchai K Techasrivichien T Suguimoto SP Ono-Kihara M. Grit is associated with lower level of depression and anxiety among University students in Chiang Mai, Thailand: a cross-sectional study. PLoS ONE. (2018) 13:e0209121. 10.1371/journal.pone.0209121 30550597 PMC6294431

[62] Lan X Ma C Radin R. Parental autonomy support and psychological well-being in tibetan and han emerging adults: a serial multiple mediation model. Front Psychol. (2019) 10:621. 10.3389/fpsyg.2019.00621 30949110 PMC6437062

[63] Beck AT Epstein N Brown G Steer RA. An inventory for measuring clinical anxiety: psychometric properties. J Consult Clin Psychol. (1988) 56:893–97. 10.1037/0022-006X.56.6.893 3204199

[64] Kohrt BA Kunz RD Koirala NR Sharma VD Nepal MK. Validation of the Nepali version of beck anxiety inventory. J Inst Med. (2003) 26:1–4.

[65] Duckworth A. Grit: The Power of Passion and Perseverance. First Scribner hardcover edition. New York, NY: Scribner (2016).

[66] Bianchi A Phillips JG. Psychological predictors of problem mobile phone use. CyberPsychol Behav. (2005) 8:39–51. 10.1089/cpb.2005.8.39 15738692

[67] Vainio MM Daukantaite D. Grit and different aspects of well-being: direct and indirect relationships via sense of coherence and authenticity. J Happiness Stud. (2016) 17:2119–47. 10.1007/s10902-015-9688-7

[68] Hill PL Burrow AL Bronk KC. Persevering with positivity and purpose: an examination of purpose commitment and positive affect as predictors of grit J Happiness Stud. (2016) 17:257–69. 10.1007/s10902-014-9593-5

[69] Baert S Vujić S Amez S Claeskens M Daman T Maeckelberghe A. Smartphone use and academic performance: correlation or causal relationship? Kyklos 73:22–46. 10.1111/kykl.12214

[70] French MT Popovici I. That instrument is lousy! In search of agreement when using instrumental variables estimation in substance use research. Health Econ. (2011) 20:127–46. 10.1002/hec.1572 20029936 PMC2888657

[71] Inchley J Currie D Young T Samdal O Torsheim T Augustson L Mathison F. editors. (2016). Growing Up Unequal: Gender and Socioeconomic Differences in Young People's Health and Well-Being: Health Behaviour in School-Aged Children (HBSC) Study: International Report from the 2013/2014 Survey. Health Policy for Children and Adolescents, no. 7. Copenhagen: World Health Organization Regional Office for Europe.

[72] WHO/CDC. Global School-Based Student Health Survey (GSHS): Nepal 2015 Fact Sheet. 2015. (2015). Available online at: http://www.who.int/ncds/surveillance/gshs/gshs_fs_nepal_2015.pdf. (accessed March 1, 2020).

[73] Kretman SE Zimmerman MA Morrel-Samuels M Hudson D. Chapter 12: adolescent violence: risk, resilience, and prevention. In: DiClemente RJ Santelli JS Crosby RA editors. Adolescent Health: Understanding and Preventing Risk Behaviors. San Francisco, CA: Jossey-Bass (2009). p. 213–32.

[74] Barber BK Stolz HE Olsen JA Collins WA Burchinal M. Parental support, psychological control, and behavioral control: assessing relevance across time, culture, and method. Monogr Soc Res Child Dev. (2005) 70:i–147. Available online at: www.jstor.org/stable/3701442 16359423 10.1111/j.1540-5834.2005.00365.x

[75] Wang MT Sheikh-Khalil S. Does parental involvement matter for student achievement and mental health in high school? Child Dev. (2014) 85:610–25. 10.1111/cdev.12153 24033259

[76] Miller AB Esposito-Smythers C Leichtweis RN. Role of social support in adolescent suicidal ideation and suicide attempts. J Adolesc Health. (2015) 56:286–92. 10.1016/j.jadohealth.2014.10.265 25561384

[77] Berkman LF Glass T. Social integration, social networks, social support, and health. in Social Epidemiology. 1st ed. Oxford: Oxford University Press (2000). p. 137–73.

[78] Kohrt BA Worthman CM. Gender and anxiety in Nepal: the role of social support, stressful life events, and structural violence. CNS Neurosci Ther. (2009) 15:237–48. 10.1111/j.1755-5949.2009.00096.x 19691543 PMC6494064

[79] Kann L McManus T Harris WA Shanklin SL Flint KH Queen B Lowry R. Youth Risk Behavior Surveillance — United States, 2017. MMWR Surv Summ. (2018) 67:1–114. 10.15585/mmwr.ss6708a1 29902162 PMC6002027

[80] MacCallum RC Austin JT. Applications of structural equation modeling in psychological research. Annu Rev Psychol. (2000) 51:201–26. 10.1146/annurev.psych.51.1.201 10751970

[81] Kline RB. Principles and Practice of Structural Equation Modeling. 4th ed. Methodology in the Social Sciences. New York, NY: The Guilford Press (2016).

[82] Fornell C Larcker DF. Structural Equation Models with Unobservable Variables and Measurement Error: Algebra and Statistics. (1981). 10.2307/3150980

[83] CommonSense. Technology Addiction: Concern, Controversy, Finding Balance. A CommonSense Research Brief. San Francisco, CA: Common Sense Media (2016).

[84] Dubicka B Theodosiou L. Technology Use and the Mental Health of Children and Young People. College Report CR225. Manchester: Royal College of Psychiatrists (2020).

[85] Orben A Przybylski AK. The association between adolescent well-being and digital technology use. Nat Hum Behav. (2019) 3:173–82. 10.1038/s41562-018-0506-1 30944443

[86] Takao M Takahashi S Kitamura M. Addictive personality and problematic mobile phone use. CyberPsychol Behav. (2009) 12:501–7. 10.1089/cpb.2009.0022 19817562

[87] Hong FY Chiu SI Huang DH. A model of the relationship between psychological characteristics, mobile phone addiction and use of mobile phones by taiwanese university female students. Comput Hum Behav. (2012) 28:2152–59. 10.1016/j.chb.2012.06.020

[88] Gillion L. Does Birth Weight Influence Grit or Can Grit Be Learned After Birth? Princeton, NJ: Working Paper.

[89] Hoeschler P Balestra S Backes-Gellner U. The development of non-cognitive skills in adolescence. Econ Lett. (2018) 163:40–45. 10.1016/j.econlet.2017.11.012

[90] American Psychiatric Association, and American Psychiatric Association, editors. Diagnostic and Statistical Manual of Mental Disorders: DSM-5. 5th ed. Washington, DC: American Psychiatric Association (2013). 10.1176/appi.books.9780890425596

[91] Deb S Strodl E Sun J. Academic stress, parental pressure, anxiety and mental health among indian high school students. Int J Psychol Behav Sci. (2015) 5:26–34. 10.5923/j.ijpbs.20150501.04

[92] Chaplin TM Gillham JE Seligman MEP. Gender, anxiety, and depressive symptoms: a longitudinal study of early adolescents. J Early Adolesc. (2009) 29:307–27. 10.1177/0272431608320125 19756209 PMC2743326

[93] Fletcher JM. Adolescent depression and educational attainment: results using sibling fixed effects. Health Econ. (2009) 19:855–71. 10.1002/hec.1526 19582699

[94] Di Domenico SI Fournier MA Ayaz H Ruocco AC. In search of integrative processes: basic psychological need satisfaction predicts medial prefrontal activation during decisional conflict. J Exp Psychol Gen. (2012) 142:967–78. 10.1037/a0030257 23067061

[95] Ryan RM Deci EL Vansteenkiste M. Autonomy and autonomy disturbances in self-development and psychopathology: research on motivation, attachment clinical process. Dev Psychopathol. (2016) 1:385–483. 10.1002/9781119125556.devpsy109

[96] Pivetta E Harkin L Billieux J Kanjo E Kuss DJ. Problematic smartphone use: an empirically validated model. Comput Hum Behav. (2019) 100:105–17. 10.1016/j.chb.2019.06.013

[97] Guerrero LR Dudovitz R Chung PJ Dosanjh KK Wong MD. Grit: a potential protective factor against substance use and other risk behaviors among Latino Adolescents. Acad Pediatr. (2016) 16:275–81. 10.1016/j.acap.2015.12.016 26796576 PMC4821776

[98] Griffin ML McDermott KA McHugh RK Fitzmaurice GM Weiss RD. Grit in patients with substance use disorders. Am J Addict. (2016) 25:652–58. 10.1111/ajad.12460 27759947 PMC5484735

[99] Borzikowsky C Bernhardt F. Lost in virtual gaming worlds: grit and its prognostic value for online game addiction: grit and online game addiction. Am J Addict. (2018) 27:433–38. 10.1111/ajad.12762 29995331

[100] Maddi SR Erwin LM Carmody CL Villarreal BJ White M Gundersen KK. Relationship of hardiness, grit, and emotional intelligence to internet addiction, excessive consumer spending, and gambling. J Posit Psychol. (2013) 8:128–34. 10.1080/17439760.2012.758306

[101] Bessey P. Preferences personality and health behaviors: results from an explorative economic experiment. Int J Health Econ Manage. (2018) 18:437–56. 10.1007/s10754-018-9236-1 29476285

[102] Duckworth AL Peterson C Matthews MD Kelly DR. Grit: perseverance and passion for long-term goals. J Pers Soc Psychol. (2007) 92:1087. 10.1037/0022-3514.92.6.1087 17547490

[103] Eskreis-Winkler L Duckworth AL Shulman EP Beal S. The grit effect: predicting retention in the military, the workplace, school and marriage. Front Psychol. (2014) 5:36. 10.3389/fpsyg.2014.00036 24550863 PMC3910317

[104] Hayes AF. Beyond baron and kenny: statistical mediation analysis in the new millennium. Commun Monogr. (2009) 76:408–20. 10.1080/03637750903310360

[105] Memon MA Cheah J Ramayah T Ting H Chuah F. Mediation analysis issues and recommendations. J Appl Struct Equat Model. (2018) 2:1–9. 10.47263/JASEM.2(1)01

[106] Zhao X Lynch JG Jr Chen Q. Reconsidering baron and kenny: myths and truths about mediation analysis. J Consum Res. (2010) 37:197–206. 10.1086/651257

[107] Aker S Sahin MK Sezgin S Oguz G. Psychosocial factors affecting smartphone addiction in University students. J Addict Nurs. (2017) 28:215–19. 10.1097/JAN.0000000000000197 29200049

[108] Chen L Yan Z Tang W Yang F Xie X He J. Mobile phone addiction levels and negative emotions among Chinese young adults: the mediating role of interpersonal problems. Comput Hum Behav. (2016) 55:856–66. 10.1016/j.chb.2015.10.030

[109] Chen B Vansteenkiste M Beyers W Boone L Deci EL Van der Kaap-Deeder J Duriez B. (2015). Basic psychological need satisfaction, need frustration, and need strength across four cultures. Motiv Emot. 39:216–36. 10.1007/s11031-014-9450-1

[110] Allen JJ Anderson CA. Satisfaction and frustration of basic psychological needs in the real world and in video games predict internet gaming disorder scores and well-being. Comput Hum Behav. (2018) 84:220–29. 10.1016/j.chb.2018.02.034

[111] Rodríguez-Meirinhos A Antolín-Suárez L Brenning K Vansteenkiste M Oliva A. A bright and a dark path to adolescents' functioning: the role of need satisfaction and need frustration across gender, age, and socioeconomic status. J Happiness Stud. (2019) 21:95–116. 10.1007/s10902-018-00072-9

[112] Yu S Levesque-Bristol C Maeda Y. General need for autonomy and subjective well-being: a meta-analysis of studies in the US and East Asia. J Happiness Stud. (2018) 19:1863–82. 10.1007/s10902-017-9898-2

[113] Seko Y Kidd S Wiljer D McKenzie K. Youth mental health interventions via mobile phones: a scoping review. Cyberpsychol. Behav. Soc. Networking 17, 591–602. 10.1089/cyber.2014.0078 25007383

[114] Oppenheim S. Should You Trust An App With Your Mental Health? Forbes. Available online at: https://www.forbes.com/sites/serenaoppenheim/2019/01/16/should-you-trust-an-app-with-your-mental-health/#38b4f1ff24b8

[115] Lear JG. Health at school: a hidden health care system emerges from the shadows. Health Aff. (2007) 26:409–19. 10.1377/hlthaff.26.2.409 17339668

[116] Brown C Dahlbeck DT Sparkman-Barnes L. (2006). Collaborative relationships: school counselors and non-school mental health professionals working together to improve the mental health needs of students. Profess Sch Counsel. 9:332–35. 10.5330/prsc.9.4.p1757020377428qv

[117] Badawy SM Radovic A. Digital approaches to remote pediatric health care delivery during the COVID-19 pandemic: existing evidence and a call for further research. JMIR Pediatr Parent. (2020) 3:e20049. 10.2196/20049 32540841 PMC7318926

[118] Serlachius A Badawy SM Thabrew H. Psychosocial challenges and opportunities for youth with chronic health conditions during the COVID-19 pandemic. JMIR Pediatr Parent. (2020) 3:e23057. 10.2196/23057 33001834 PMC7553787

[119] Calvo RA Peters D. Positive Computing: Technology for Wellbeing and Human Potential. Boston, MA: The MIT Press (2014). 10.7551/mitpress/9764.001.0001

[120] Karlan P Bankman J. Can Technology Solve the Mental Health Crisis? With Guests. Ruzek J, Harned Z, Darcy A. Standford Legal (nd). Available online at: https://law.stanford.edu/stanford-legal-on-siriusxm/can-technology-solve-the-mental-health-crisis-with-guests-dr-joe-ruzek-and-zach-harned/. (accessed March 1, 2020).

[121] Muzaffar M. The Mental Health App That Checks In on You Like a Friend. The Daily Dose (2019). Available online at: https://www.ozy.com/good-sht/meet-youper-your-personal-therapist/95935/. (accessed March 1, 2020).

[122] Perski O Blandford A Ubhi HK West R Michie S. Smokers' and drinkers' choice of smartphone applications and expectations of engagement: a think aloud and interview study. BMC Med Inform Decis Mak. (2017) 17:25. 10.1186/s12911-017-0422-8 28241759 PMC5329928

[123] Perski O Blandford A West R Michie S. Conceptualising engagement with digital behaviour change interventions: a systematic review using principles from critical interpretive synthesis. Transl Behav Med. (2017) 7:254–67. 10.1007/s13142-016-0453-1 27966189 PMC5526809

[124] Chen B Liu F Ding S Ying X Wang L Wen Y. Gender differences in factors associated with smartphone addiction: a cross-sectional study among medical college students. BMC Psychiatry. (2017) 17:341. 10.1186/s12888-017-1503-z 29017482 PMC5634822

[125] Maharjan RK Plan Nepal (Organization) Save the Children (U.S.), World Vision International Nepal, editors. Child Marriage in Nepal: Research Report. Kathmandu: Plan Nepal : Save the Children, Nepal Country Office : World Vision International Nepal (2012).

[126] Fatusi AO Hindin MJ. Adolescents and youth in developing countries: health and development issues in context. J Adolesc. (2010) 33:499–508. 10.1016/j.adolescence.2010.05.019 20598362

[127] Chisholm D Sweeny K Sheehan P Rasmussen B Smit F Cuijpers P. Scaling-up treatment of depression and anxiety: a global return on investment analysis. Lancet Psychiatry. (2016) 3:415–24. 10.1016/S2215-0366(16)30024-4 27083119

[128] Blanchflower D. Is Happiness U-Shaped Everywhere? Age and Subjective Well-Being in 132 Countries. w26641. Cambridge, MA: National Bureau of Economic Research (2020). 10.3386/w26641

[129] Blanchflower D. Unhappiness and Age. w26642. Cambridge, MA: National Bureau of Economic Research (2020). 10.3386/w26642

