# Supplementary material for: mHealth: Potentials and Risks for Addressing Mental Health and Well-Being Issues Among Nepali Adolescents
Source: Front Public Health. 2021 Apr 23;9:563515. doi: 10.3389/fpubh.2021.563515 (PMC8102693; doi:10.3389/fpubh.2021.563515)
Supplement: Supplementary file 1 [file Data_Sheet_1.docx]

**APPENDIX**

1. **Additional Details on Variables (Table A1)**
2. **Descriptive Statistics by Gender (Table A2)**
3. **Correlation Matrix of Key Scale Variables (Table A3)**
4. **Sensitivity Analyses (Tables A4 & A5)**

**APPENDIX A - Table A1: Additional Details on Variables**

| VARIABLES | DESCRIPTION |
| --- | --- |
| OUTCOME VARIABLES |  |
| *Anxiety* | Beck Anxiety Inventory (BAI) ^†^, a validated survey instrument, where symptoms evaluated include numbness/tingling, feeling hot, leg wobbliness, inability to relax, fear of the worst, dizzy/lightheaded, heart pounding/racing, unsteady, terrified/afraid, nervous, choking, hands trembling, shakiness, breathing difficulty, fear of dying, & being scared, where 0= No Experience of Symptom & 3= Severe Experience of Symptom over the last few days |
| *Grit Score* | Duckworth Grit Scale^‡^*^,^* a validated survey instrument, which queries agreement with statements such as “I often set a goal but later chose to pursue a different one.” and “My interests change from year to year.”, where 1= Strongly Agree & 5= Strongly Disagree, along with statements such as “Setbacks don’t discourage me. I don’t give up easily.” and “ I have overcome setbacks to conquer an important challenge.”, where 1=Strongly Disagree & 5= Strongly Agree |
|  |  |
| Key Explanatory Variables | |
| *Problematic Mobile Phone Use (PMPU)* | Validated survey instrument^§^ which asks agreement on statements such as “I can never spend enough time on my phone”, “I have tried to hide from others how much time I spend on my mobile phone”, & “ I have frequent dreams about the mobile phone”, where 1= Strongly Disagree & 5=Strongly Agree |
| *Bullying* | Summation index composed of “Yes” answers to questions on being physically hurt within the prior year, being bullied in school the prior month, & being bullied outside of school the prior month. |
|  |  |
| Socio-Cultural Environmental Pressures Controlled For | |
|  |  |
| *Academic Pressures* | \| Latent variable measured through indicators capturing agreement with statements regarding experiences over the last month of being worried a lot about exam scores, having teachers that are too controlling, a school environment that is extremely competitive, & belief that success in school will determine life success , where 1= Strongly Agree & 5= Strongly Disagree \| \| --- \| |
| *Family Environment* | Latent variable measured through indicators capturing agreement with statements regarding experiences over the last month of parents checking the contents of respondent’s mobile phone, having been physically hurt in his/her household, have been punished by parents for bad grades/exam scores & that women should tolerate violence in order to keep familial harmony , where 1= Strongly Agree & 5= Strongly Disagree |
| *Social Support* | Latent variable measured through binary indicators to having someone outside family to borrow money from, stay with in case of a problem, confide in about violence, assist if someone is harassing respondent, a place to meet same sex friends, & membership in a social/cultural club or youth group. |
| Instruments (For Potentially Endogenous PMPU) | |
| *Phone Cost* | Open-ended query on the cost of mobile phone in Nepali Rupees (divided by 1,000) |
| *Friend’s PMPU* | Reworked instrument asks agreement on statements such as “My close friends are on their mobile phone when they should be doing other things, and it causes problems.”, “My close friends try to hide from others how much time they spend on the mobile phone.”, & “You complain about your friends’ use of the mobile phone.” where 1= Strongly Disagree & 5=Strongly Agree |

†( Beck, 1988) & (Kort, 2003)

‡ (Duckworth, 2016)

§ (Biancchi & Philips, 2005)

**APPENDIX B - Table A2: Descriptive Statistics by Gender**

| VARIABLES | FEMALES | | MALES | | Min/Max |
| --- | --- | --- | --- | --- | --- |
|  | Mean | Standard Deviation | Mean | Standard Deviation |  |
| OUTCOME VARIABLES |  |  |  |  |  |
| *Anxiety (A)* | 14.51 | 7.97 | 12.24 | 7.91 | 0/48 |
| *Grit Score (G)* | 3.27 | 0.50 | 3.26 | 0.50 | 1.8/5 |
| KEY EXPLANATORY VARIABLES | | | | | |
| *Problematic Mobile Phone Use (PMPU)* | 83.95 | 20.55 | 89.21 | 21.40 | 27/135 |
| *Bullying (B)* | 0.34 | 0.68 | 0.37 | 0.73 | 0/3 |
| Physically Hurt Prior Year | 0.099 | - | 0.164 | - | 0/1 |
| Bullied at School | 0.112 | - | 0.101 | - | 0/1 |
| Bullied Outside School | 0.128 | - | 0.104 | - | 0/1 |
| SOCIO-CULTURAL ENVIRONMENTAL FACTORS | | | | | |
| *Academic Pressures (AP*)* |  |  |  |  |  |
| Worry About Exam Scores | 4.10 | 1.27 | 3.97 | 1.33 | 1/5 |
| Teachers Too Controlling | 3.63 | 1.43 | 3.72 | 1.34 | 1/5 |
| School Competitive | 3.99 | 1.21 | 3.74 | 1.34 | 1/5 |
| School Success is Life Success | 4.28 | 1.17 | 4.01 | 1.35 | 1/5 |
| *Family Environment (FE*)* |  |  |  |  |  |
| Parents Check Phone | 3.29 | 1.62 | 3.07 | 1.62 | 1/5 |
| Physically Hurt in Home | 2.40 | 1.56 | 2.68 | 1.58 | 1/5 |
| Punished for Bad Grades | 2.59 | 1.66 | 2.69 | 1.58 | 1/5 |
| Women Tolerate Violence | 2.50 | 1.66 | 2.72 | 1.61 | 1/5 |
| *Social Support (SS*)* |  |  |  |  |  |
| Borrow Money | 0.734 | - | 0.732 | - | 0/1 |
| Stay With | 0.694 | - | 0.718 | - | 0/1 |
| Confide in About Violence | 0.618 | - | 0.678 | - | 0/1 |
| Help with Harassment Situation | 0.648 | - | 0.658 | - | 0/1 |
| Place Meet Same Sex Friends | 0.418 | - | 0.487 | - | 0/1 |
| Member of Club/Youth Group | 0..355 | - | 0.480 | - |  |
| INSTRUMENTS (FOR *PMPU*) |  |  |  |  |  |
| *Phone Cost (PC)* | 18.27 | 15.37 | 19.86 | 16.25 | 0.2/110 |
| *Phone Cost Sq. (PC2)* | 569.06 | 1431.13 | 656.63 | 1315.51 | 0.04/12100 |
| *Friend’s PMPU (FPMPU)* | 19.42 | 5.71 | 20.76 | 5.47 | 6/30 |
| ADDITIONAL CONTROLS |  |  |  |  |  |
| *Age (X1)* | 17.41 | 1.12 | 17.78 | 1.23 | 15/25 |
| *Rural (X3)* | 0.576 | - | 0.403 | - | 0/1 |

**APPENDIX C - Table A3: Correlation Matrix of Key Scale Variables (Table A3)**

|  | **Anxiety** | **Grit** | **PMPU** | **Bullying** |
| --- | --- | --- | --- | --- |
| **Anxiety** | 1.00 | - | - | - |
| **Grit** | -0.237*** | 1.00 | - | - |
| **PMPU** | 0.256*** | -0.398*** | 1.00 | - |
| **Bullying** | 0.191*** | -0.227*** | 0.193*** | 1.00 |

*** p<0.01, ** p<0.05, * p<0.1

**APPENDIX D -** **Sensitivity Analysis**

To facilitate in determining the robustness of the findings from our main structural modelling estimation, which incorporated the ability to account for measurement error in certain latent measures of key adolescent-life pressure points, several sensitivity analyses were performed. These additional estimation approaches do not allow one to account for the measurement error of the latent constructs. Each of the three constructs were operationalized through summing the values of the respective indicators detailed in Table 2.2. Due to collinearity issues, the use of the demographic control *X3* (e.g. rural binary indicator), was also removed in these analyses.

The first sensitivity analysis was a baseline estimation of ordinary least squares (OLS), which would ignore any potential endogeneity to be found between our key technology variable (*PMPU)* and either of the wellbeing outcome variables. As seen in columns one and five of Table A1, there is a statistically significant positive (negative) impact of *PMPU* on anxiety (grit), with impacts of bullying mirroring these results in terms of sign and significance. Females are indicated, just as with structural equation modeling (SEM), to have a higher likelihood of exhibiting more anxiety symptoms. Our measure of academic pressure in this context only has a significant (positive) association with grit, while social support is shown to have a statistically significant negative effect on anxiety. This latter outcome is the major departure from the results found through SEM. We address speculation as to this outcome in the main text of this work. There is indication from literature of potential (econometric) endogeneity between the key technology/mediating variable, *PMPU,* and the two wellbeing outcomes, as referenced in the body of this work. This required that we also attempt a number of instrumental variable estimation methods under this regression-oriented estimation framework testing the sensitivity of our results. Determination of the best instruments required initial exploration of studies examining similar outcome and explanatory variables.

Using the National Longitudinal Study of Adolescent Health (AddHealth) database, researchers examining the impact of suicidal thoughts/attempts on likelihood of engaging in school used the suicidal behavior of friends as an instrument to account for reverse causality concerns (Tekin and Markowitz 2008). As applied to this work, such an approach would be based on the assumption that the perception of friends’ use/overuse of cell phones impacts one’s own use/overuse of mobile phones, but does not impact wellbeing outcomes directly. Similarly, prices have also been a common instrument used in economic studies investigating substance abuse (Fang, Ali, and Rizzo 2009; Amialchuk, Bornukova, and Ali 2018; French and Popovici 2011). Thus, we can capture the prices of cell phone, based on the assumption that the cost of a phone will impact its use/overuse, but will not directly affect wellbeing outcomes such as anxiety and grit.

Using the above instruments, estimations were undertaken using traditional two-stage least squares (2SLS) and with two-step feasible generalized method of moment (GMM2) estimation. The latter estimator is more efficient relative to 2SLS due to the use of an optimal weighting matrix and relaxation of the *iid* assumption (Hayashi 2000). Results are shown in Table A1, columns (2)–(3) and (6)-(7) as they pertain to the outcomes of anxiety and grit, respectively. Results in terms of sign and significance from these IV techniques are consistent with those found from OLS, where the only departure from the SEM results is the significant negative impact of our measure of social support on anxiety. Compared to those estimates from OLS, these coefficient estimates are of similar magnitude to those found with OLS, which would provide support for an argument that simultaneity between anxiety and problematic phone use is not a concern. In contrast, the OLS coefficient for *PMPU*’s effect on grit is attenuated towards zero in comparison to the IV estimates, supporting an argument that there is potential bias caused from endogeneity for this estimation equation. Such conclusions match with the results found via our SEM approaches that the covariances between *PMPU* and *A* are insignificant, while those between *PMPU* and *G* are significant. These conclusions are also supported by the test statistics reported at the bottom of Table A1.

Appropriate application of instrument variable techniques requires determination of both the satisfaction of the exclusion restriction and strength of the relevance condition. With respect to the strength of our instrumental variables (i.e. their predictive power concerning overuse/problematic use of mobile phones), we conduct F-tests of their joint significance in the first stage of the 2SLS regressions. We also report the Sargan/Hansen J over-identification test. With respect to the exogeneity of our instrumental variables, we present the Hausman test statistic. One can see that the Hausman tests for exogeneity of PMPU are not rejected in the case where anxiety is the outcome variable, while they are rejected with marginal significance (at the 90% level) in the case where grit is the outcome variable. Regardless of outcome variable, however, the Sargan/Hansen J statistics are not rejected, supporting the null hypothesis that the instruments are valid (or more appropriately, that the exclusion restriction is met), should *PMPU* be deemed as leading to an endogeneity problem. Additionally, the strength of our set of instruments is supported by F-tests in the first stage equation being larger than 10 (Greene 2003).

The final (limited information) technique undertaken was to re-estimate our 2SLS models, with an IV technique that has been developed which does not require using any external instruments. This Lewbel technique (2012) has been used in examinations of smoking and subjective wellbeing (happiness and depression), along with work examining the interplay of alcohol consumption and depression (Awaworyi Churchill and Farrell 2017a; 2017b). The premise is to internally generate instruments in an approach similar to 2SLS, when external instruments are unavailable or weak. Even in the presence of potentially strong instruments, the use of the Lewbel technique is believed to increase the strength of the estimation with truly exogenous instruments. One constructs internal instruments from the residuals of auxiliary equations that are multiplied by included exogenous variables in their mean-centered form. Identification with this approach stems from heteroskedastic covariance restrictions. As shown in the final columns ((4) and (8)) of Table A1, the results of including these additional instruments are unchanged from those found with the prior two IV techniques.

Given that limited information methods such as 2SLS and GMM2 do not properly account for the (potential) correlation between the two wellbeing outcomes themselves, we also undertook the full-information estimation technique of three-stage least squares (3SLS), results of which can be found in Table A2. To mirror the approach used in our main structural modeling analyses, we present two versions of the estimation: one where we include demographic controls in the equation for *PMPU*, and one where we do not (first column). Results here again indicate the same sign and significance for all key variables. Across the board, the magnitude of parameter estimates are smaller than those found with IV or SEM techniques. Additionally, the strength of a significant positive effect of social support on anxiety is smaller.

In total, these sensitivity analyses we believe provide good support for the findings of our structural equation estimation results. They reemphasize the importance of monitoring problematic mobile phone use and bullying to avoid deleterious wellbeing outcomes. Further, they provide empirical support for the BPNT framework and the role that need thwarting contexts play in reducing overall wellbeing.

**REFERENCES**

Amialchuk, Aliaksandr, Kateryna Bornukova, and Mir M. Ali. 2018. “Will a Decline in Smoking Increase Body Weights? Evidence from Belarus.” *Eastern Economic Journal* 44 (2): 190–210. https://doi.org/10.1057/s41302-016-0071-0.

Awaworyi Churchill, S., and L. Farrell. 2017a. “Alcohol and Depression: Evidence from the 2014 Health Survey for England.” *Drug and Alcohol Dependence* 180 (November): 86–92. https://doi.org/10.1016/j.drugalcdep.2017.08.006.

———. 2017b. “Investigating the Relationship between Smoking and Subjective Welfare.” *Journal of Behavioral and Experimental Economics* 71 (December): 1–12. https://doi.org/10.1016/j.socec.2017.08.003.

Fang, Hai, Mir M. Ali, and John A. Rizzo. 2009. “Does Smoking Affect Body Weight and Obesity in China?” *Economics & Human Biology* 7 (3): 334–50. https://doi.org/10.1016/j.ehb.2009.07.003.

French, Michael T., and Ioana Popovici. 2011. “That Instrument Is Lousy! In Search of Agreement When Using Instrumental Variables Estimation in Substance Use Research.” *Health Economics* 20 (2): 127–46. https://doi.org/10.1002/hec.1572.

Greene, William H. 2003. *Econometric Analysis*. 5th ed. Upper Saddle River, NJ: Prentice-Hall.

Hayashi, Fumio. 2000. *Econometrics*. Princeton, N.J: Princeton University Press.

Lewbel, Arthur. 2012. “Using Heteroscedasticity to Identify and Estimate Mismeasured and Endogenous Regressor Models.” *Journal of Business & Economic Statistics* 30 (1): 67–80. https://doi.org/10.1080/07350015.2012.643126.

Tekin, Erdal, and Sara Markowitz. 2008. “The Relationship between Suicidal Behavior and Productive Activities of Young Adults.” *Southern Economic Journal* 75: 300–331.

**Table A3: IV Results for Adolescent Life Influences on Health Outcomes of Anxiety & Grit**

|  | **Anxiety** | | | | **Grit** | | | |
| --- | --- | --- | --- | --- | --- | --- | --- | --- |
| VARIABLES | OLS  (1) | 2SLS  (2) | GMM2  (3) | Lewbel^¶^  (4) | OLS  (5) | 2SLS  (6) | GMM2  (7) | Lewbel^¶^  (8) |
|  |  |  |  |  |  |  |  |  |
| *PMPU*^†^ | 0.0946*** | 0.0929*** | 0.1000*** | 0.0925*** | -0.00895*** | -0.0135*** | -0.0139*** | -0.0134*** |
|  | (0.0118) | (0.0277) | (0.0252) | (0.0285) | (0.00135) | (0.00195) | (0.00178) | (0.00176) |
| *B* | 1.918*** | 1.977*** | 1.772*** | 1.978*** | -0.109*** | -0.0918*** | -0.0717*** | -0.0921*** |
|  | (0.180) | (0.338) | (0.305) | (0.339) | (0.0209) | (0.0261) | (0.0189) | (0.0268) |
| *AP** | 0.0282 | 0.0320 | -0.0237 | 0.0326 | 0.0133** | 0.0223*** | 0.0260*** | 0.0222** |
|  | (0.0744) | (0.0804) | (0.0723) | (0.0765) | (0.00608) | (0.00850) | (0.00773) | (0.00799) |
| *FE** | 0.0520 | 0.0226 | 0.0431 | 0.0231 | -0.00983* | -0.00224 | -0.00451 | -0.00236 |
|  | (0.0626) | (0.0629) | (0.0615) | (0.0718) | (0.00462) | (0.00477) | (0.00392) | (0.00453) |
| *SS** | -0.486*** | -0.398*** | -0.317** | -0.398** | 0.0200 | 0.0276* | 0.0181 | 0.0276 |
|  | (0.125) | (0.143) | (0.129) | (0.150) | (0.0190) | (0.0153) | (0.0124) | (0.0163) |
| *Age* | 0.497* | 0.588*** | 0.497*** | 0.589** | -0.00835 | -0.00342 | -0.00765 | -0.00348 |
|  | (0.240) | (0.220) | (0.112) | (0.230) | (0.0150) | (0.0157) | (0.0150) | (0.0163) |
| *Female* | 2.509*** | 2.660*** | 2.330*** | 2.658*** | -0.0352 | -0.0585 | -0.0405 | -0.0581 |
|  | (0.743) | (0.735) | (0.617) | (0.759) | (0.0444) | (0.0437) | (0.0403) | (0.0465) |
| *Constant* | -4.886 | -6.637 | -5.118 | -6.622 | 4.076*** | 4.141*** | 4.258*** | 4.138*** |
|  | (5.326) | (5.571) | (3.190) | (5.851) | (0.261) | (0.264) | (0.242) | (0.282) |
|  |  |  |  |  |  |  |  |  |
| N | 527 | 485 | 485 | 485 | 500 | 459 | 459 | 459 |
| R^2^ | 0.143 | 0.152 | 0.150 | 0.152 | 0.203 | 0.164 | 0.154 | 0.165 |
| AIC | 3619.81 | 3304.13 | 3305.10 | 3304.14 | 633.07 | 596.28 | 601.63 | 595.68 |
| BIC | 3653.95 | 3337.60 | 3338.57 | 3337.61 | 666.79 | 629.32 | 634.67 | 628.71 |
|  |  |  |  |  |  |  |  |  |
| F-Value First Stage |  | 39.46 | 39.46 |  |  | 42.87 | 42.87 |  |
| Sargan/Hansen J^‡^ |  | 2.761 | 2.761 |  |  | 1.380 | 1.380 |  |
| (p-value) |  | (0.251) | (0.251) |  |  | (0.502) | (0.502) |  |
| Hausman^§^ |  | 0.104 | 0.104 |  |  | 3.750 | 3.750 |  |
| (p-value) |  | (0.747) | (0.747) |  |  | (0.053) | (0.053) |  |

Standard Errors Clustered at the School-Grade Level; *** p<0.01, ** p<0.05, * p<0.1

†Instrumented with PhoneCost, PhoneCostSQ, FriendsPMPU

‡ H0: Instruments Are Valid

§H0: Endog. Var Are Actually Exog.

¶ Instruments used are those generated internally from the data & external instruments, where use of clustered SE’s doesn’t allow for appropriate testing of endogeneity or overID

**Table A4: 3SLS Results for Adolescent Life Influences on Health Outcomes of Anxiety & Grit**

|  | **No Demographic Controls** | | | **With Demographic Controls** | | |
| --- | --- | --- | --- | --- | --- | --- |
| VARIABLES | Grit | Anxiety | PMPU Score | Grit | Anxiety | PMPU Score |
|  |  |  |  |  |  |  |
| *PMPU* | -0.0122*** | 0.0895*** |  | -0.0122*** | 0.0894*** |  |
|  | (0.00206) | (0.0334) |  | (0.00206) | (0.0334) |  |
| *B* | -0.0853*** | 1.542*** | 1.225 | -0.0852*** | 1.542*** | 1.246 |
|  | (0.0286) | (0.464) | (0.977) | (0.0286) | (0.464) | (0.975) |
| *AP** | 0.0203*** | 0.0102 | 0.752*** | 0.0205*** | 0.0105 | 0.797*** |
|  | (0.00697) | (0.113) | (0.223) | (0.00697) | (0.113) | (0.224) |
| *FA** | -0.00518 | 0.0180 | 0.723*** | -0.00517 | 0.0180 | 0.720*** |
|  | (0.00547) | (0.0886) | (0.170) | (0.00547) | (0.0886) | (0.170) |
| *SS** | 0.0312** | -0.415* | 0.229 | 0.0308** | -0.415* | 0.150 |
|  | (0.0132) | (0.214) | (0.455) | (0.0132) | (0.214) | (0.456) |
| *PC* |  |  | 0.392*** |  |  | 0.381*** |
|  |  |  | (0.119) |  |  | (0.119) |
| *PC2* |  |  | -0.00350** |  |  | -0.00337** |
|  |  |  | (0.00137) |  |  | (0.00136) |
| *FPMPU* |  |  | 1.897*** |  |  | 1.887*** |
|  |  |  | (0.137) |  |  | (0.139) |
| *Age* | 0.00122 | 0.546** |  | -0.000983 | 0.544** | -0.459 |
|  | (0.0164) | (0.269) |  | (0.0166) | (0.269) | (0.579) |
| *Female* | -0.0504 | 2.945*** |  | -0.0616 | 2.932*** | -2.321* |
|  | (0.0408) | (0.671) |  | (0.0414) | (0.671) | (1.407) |
| *Constant* | 3.993*** | -4.881 | 22.57*** | 4.038*** | -4.832 | 31.75*** |
|  | (0.315) | (5.157) | (4.173) | (0.318) | (5.158) | (10.80) |
|  |  |  |  |  |  |  |
| N | 522 | 522 | 522 | 522 | 522 | 522 |
| R^2^ | 0.176 | 0.133 | 0.437 | 0.176 | 0.133 | 0.440 |
| AIC (Overall) | 8595.99 | | | 8596.93 | | |
| BIC (Overall) | 8698.18 | | | 8707.63 | | |

Standard errors in parentheses; *** p<0.01, ** p<0.05, * p<0.1
